# Prognostic Associations and Functional Implications of Angiogenesis-Related miRNA Variants in Ischemic Stroke

**DOI:** 10.3390/cells14171389

**Published:** 2025-09-05

**Authors:** Chang Soo Ryu, Kee-Ook Lee, Eun Ju Ko, Hyeon Woo Park, Jae Hyun Lee, Ok Joon Kim, Nam Keun Kim

**Affiliations:** 1 Department of Life Science, Graduate School, CHA University, 335 Pangyo-ro, Bundang-gu, Seongnam 13488, Republic of Korea; regis2040@nate.com (C.S.R.); ejko05@naver.com (E.J.K.); aabb1114@naver.com (H.W.P.); athe7a@naver.com (J.H.L.); 2 Division of Life Sciences, College of Life Sciences, CHA University, 335 Pangyo-ro, Bundang-gu, Seongnam 13488, Republic of Korea; 3 Department of Neurology, CHA Bundang Medical Center, School of Medicine, CHA University, Seongnam 13496, Republic of Korea; niceiatros@cha.ac.kr

**Keywords:** angiogenesis, microRNA, ischemic stroke, polymorphism, prognosis

## Abstract

Ischemic stroke is a multifactorial cerebrovascular disease that remains a leading cause of long-term disability and mortality worldwide. Despite advances in acute treatment, recurrence rates remain high, and nearly half of survivors experience persistent neurological deficits. Therefore, identifying genetic biomarkers that contribute to early diagnosis, risk prediction, and therapeutic improvement is increasingly important. MicroRNAs, small non-coding RNAs involved in gene regulation, have been recognized for their critical roles in vascular development and angiogenesis. This study investigated the association between angiogenesis-related miRNA gene polymorphisms and ischemic stroke risk using a population-based case–control design. Genotyping and statistical analysis revealed that *miR-21* rs13137 A > T and *miR-126* rs4636297 G > A were significantly associated with stroke susceptibility. The TT genotype of *miR-21* rs13137 demonstrated a protective effect (*p* = 0.019); the AA genotype of *miR-126* rs4636297 was associated with increased risk (*p* = 0.006), along with its dominant model (*p* = 0.007). Additionally, deep learning models were utilized to evaluate gene–gene and gene–environment interactions, enhancing predictive accuracy and identifying synergistic effects between miRNA polymorphisms and clinical risk factors. In summary, specific miRNA variants may serve as novel biomarkers for ischemic stroke, providing valuable insight into genetic susceptibility and supporting the advancement of precision medicine strategies.

## 1. Introduction

Ischemic stroke is a disease characterized by brain damage resulting from insufficient blood flow, stenosis, or occlusion of the cerebral blood vessels responsible for delivering oxygen and nutrients to the brain [1]. It occurs in approximately 80% of all strokes, including hemorrhagic strokes, and remains a fatal disease, ranking as the third leading cause of death annually in Korea [1,2,3,4]. Cerebrovascular disease is a multifactorial condition, rather than a single factor–derived pathology. It is influenced by genetic predisposition and multiple environmental factors, including advanced age, alcohol abuse, blood coagulation disorders, diabetes mellitus, a personal or family history of stroke, hypertension and metabolic syndrome [5,6,7,8,9]. Previous studies have identified hypertension, diabetes mellitus, hyperlipidemia, and smoking as key risk factors for ischemic stroke [9,10,11]. Pharmacological treatments, such as recombinant tissue plasminogen activator, are typically administered during the acute phase (within 4 to 6 h of onset). In the chronic phase, which constitutes the majority of stroke cases, non-pharmacological interventions (e.g., stent placement) are often utilized. The recurrence rate is reportedly up to 43%; the mortality rate within 10 years after onset ranges from approximately 17% to 93%, depending on age, and the average rate is around 45%. Additionally, approximately 50% of survivors experience persistent physical disabilities, along with conditions such as dementia and depression [12,13,14,15]. Emerging evidence indicates that stem cell-derived exosomes and exosomal biomarkers represent promising therapeutic options and genetic markers after ischemic stroke, based on mouse model studies [16,17,18,19]. Overall, there is an urgent need for new treatments capable of minimizing the neurological impairments that frequently occur after stroke.

There is some evidence that the identification of key biomarkers can aid diagnosis and therapeutic management of ischemic stroke patients [20,21]. However, existing biomarkers have shown limitations, emphasizing the importance of new biomarkers for prognostic and diagnostic prediction in ischemic stroke [22,23]. Previous research in our laboratory has facilitated the identification of multiple potential biomarkers for diagnostic prediction in ischemic stroke [24,25,26,27,28,29]. In particular, one study of microRNA (miRNA) polymorphisms revealed a significant association between ischemic stroke risk and specific polymorphisms [24]. The study indicated that *miR-34a* and *miR-130a* were significantly associated with ischemic stroke risk and mortality. Another study demonstrated that these miRNA polymorphisms were correlated with mortality after ischemic stroke [28]. Based on these findings, we aimed to focus on lesser-studied miRNAs in ischemic stroke; we hypothesized that these genes could serve as novel diagnostic biomarkers and influence angiogenic function after ischemic injury.

Recent miRNA-related studies across various conditions have identified miRNAs as biological regulators of angiogenesis and neurogenesis [30,31,32]. In particular, a role for miRNAs has been identified in downregulating the expression of specific cell-cycle genes [33]. miRNAs bind to various regions within the 3′-untranslated regions (3′-UTRs) of target gene mRNAs, thereby influencing the translational capacity of these genes [34]. Previous studies have shown that miRNAs regulate the expression of their target genes and affect the pathogenesis of multiple diseases, including ischemic stroke [31,35,36,37,38]. These investigations have confirmed that miRNAs substantially contribute to key physiological and pathological processes [39,40,41,42]. There is increasing recognition of the relationship between aberrant miRNA expression and the mechanisms of angiogenesis after ischemic stroke [38,43,44]. Studies of critical post-stroke mechanisms, including angiogenesis, neurogenesis, and neuroprotection, have demonstrated that miRNAs may play vital roles in the pathophysiology of ischemic stroke [17,44,45,46,47]. Moreover, several studies have confirmed that the therapeutic delivery of miRNAs represents a potential treatment option for stroke patients, further supporting the importance of angiogenesis in stroke recovery [48,49]. Additionally, stem cell-derived exosomes and exosomal miRNAs may serve as suitable therapeutic and biomarker candidates in the context of ischemic stroke angiogenesis [47]. Consequently, various expression studies of miRNAs have supported the potential of genetic biomarkers in ischemic stroke and their roles in modulating angiogenesis after ischemic injury [18,38,44,50,51,52].

Previous studies of specific miRNAs, including *miR-21*, *miR-26a*, *miR-107*, *miR-124-1*, and *miR-126*, have identified their ability to influence the expression levels of angiogenesis-related target genes and have demonstrated the potential for genetic biomarkers to regulate angiogenesis in the context of ischemic stroke [50,52,53,54,55,56,57,58]. Specifically, one study revealed that exosomal *miR-21* enhances angiogenic function in ischemic stroke mouse models [59]. Another study showed that *miR-26a* promotes angiogenesis in cerebral infarction rat models through activation of the phosphoinositide 3-kinase/protein kinase B and mitogen-activated protein kinase/extracellular signal-regulated kinase pathways [60]. Additional research has indicated that *miR-107* modulates *Dicer-1* expression during post-stroke angiogenesis [61]. Similarly, there is evidence that *miR-124* regulates angiogenesis in peripheral arterial disease by modulating *STAT3* expression [62]. Finally, a study of *miR-126* demonstrated that its modulation mitigates brain injury and accelerates functional recovery after stroke [47].

Using the National Center for Biotechnology Information database, this study investigated less well-known angiogenesis-related gene polymorphisms associated with ischemic stroke, revealing several novel miRNA gene polymorphisms. Consequently, we designed a genetic epidemiological study and confirmed significant associations of six miRNA gene polymorphisms (*miR-21* rs1292037 T > C, rs13137 A > T; *miR-26a* rs7372209 C > T; *miR-107* rs2296616 A > G; *miR-124-1* rs531564 G > C; *miR-126* rs4636297 G > A) with ischemic stroke in a Korean population. Furthermore, we analyzed differences in expression of these miRNAs’ target genes according to their respective polymorphisms, based on post-transcriptional regulatory mechanisms. Additionally, we sought to identify significant differences in exosomal miRNA expression between ischemic stroke patients and controls through small RNA sequencing. To our knowledge, this study is the first to demonstrate that these six polymorphisms influence the susceptibility of Korean individuals to ischemic stroke.

## 2. Materials and Methods

### 2.1. Study Approval

The Institutional Review Board of CHA Bundang Medical Center approved and reviewed the protocols of all studies on 6 November 2013 (IRB number: 2013-09-073), following the Declaration of Helsinki recommendation. This study has obtained informed consent from all participants.

### 2.2. Study Population Group

Blood samples utilized in this study were obtained from 520 patients diagnosed with ischemic stroke and 400 control individuals. All participants were recruited from the Department of Neurology at CHA Bundang Medical Center, CHA University, during the period from 2000 to 2010. Ischemic stroke subtypes were classified by two board-certified neurologists based on clinical manifestations and neuroimaging findings, according to the Trial of Org 10172 in Acute Stroke Treatment (TOAST) classification system [63,64,65]. The subtypes were defined as follows: (1) large-artery disease (LAD), characterized by an infarct lesion measuring ≥15 mm in diameter on magnetic resonance imaging (MRI) with evidence of significant stenosis (>50%) in a major cerebral artery; (2) small-vessel disease (SVD), defined by infarcts measuring between 5 mm and <15 mm in diameter, presenting with classic lacunar syndromes, and lacking signs of cortical dysfunction or cardioembolic sources; (3) cardio-embolism (CE), referring to arterial occlusions presumed to be caused by emboli originating from the heart; and (4) undetermined etiology, where the cause could not be confidently identified or where multiple potential causes coexisted. The distribution of stroke subtypes within the patient population was as follows: LAD, 39.2% (n = 204); SVD, 28.5% (n = 148); CE, 11.2% (n = 58); and undetermined, 19.4% (n = 101). Controls were drawn from subjects visiting our hospitals during the same period for health examinations, including biochemical testing, electrocardiograms, and brain MRIs. None of the control subjects had a history of cerebrovascular disease or myocardial infarction. The exclusion criteria applied to the control group were identical to those used for the stroke group. Hypertension, diabetes mellitus, and hyperlipidemia were defined according to standard clinical criteria, including diagnosis based on laboratory findings or the use of relevant medications.

### 2.3. Genotyping of the Six miRNA Polymorphisms

Genomic DNA was extracted from peripheral blood leukocytes using the G-DEX II Blood Extraction Kit (iNtRON Biotechnology, Seongnam, Republic of Korea). A total of six microRNA (miRNA) polymorphisms were analyzed using two genotyping methods. *miR-124-1* rs531564 G > C polymorphism was confirmed using polymerase chain reaction-restriction fragment length polymorphism (PCR-RFLP). The primer sequences for this single-nucleotide polymorphism (SNP) were as follows: *miR-124-1* rs531564 G > C, forward 5′-CTG TGA CAG ACA GGG GCT TAG-3′ and reverse 5′-AAA CAC AGT CAC GGA GGA AGG-3′. Enzymatic digestion of PCR products was performed using *Bsm*AI restriction enzymes (New England BioLabs, Ipswich, MA, USA) at 37 °C for 16–24 h. The remaining *miR-21* rs1292037 T > C, rs13137 A > T, *miR-26a* rs7372209 C > T, *miR-107* rs2296616 A > G, and *miR-126* rs4636297 G > A were genotyped using real-time PCR and further validated via PCR-based DNA sequencing. Moreover, detailed population genotype frequencies for all analyzed miRNA SNPs are presented in Tables S1–S4.

### 2.4. Determination of Homocysteine, Folate, and High-Density Lipoprotein-Cholesterol Levels

Specimens were collected to measure total homocysteine and folate levels within 48 h after the onset of a stroke. The total homocysteine (tHcy) concentration in the plasma was measured using a fluorescent polarizing immunoassay (FPIA) with IMx system (Abbott Laboratories, Chicago, IL, USA). A radioassay kit (ACS: 180; Bayer, Tarrytown, NY, USA) measured the concentration of plasma folate levels. Lastly, the enzymatic colorimetric methods of commercial reagent sets (TBA 200FR NEO, Toshiba Medical Systems, Tochigi, Japan) measured the high-density lipoprotein-cholesterol (HDL-C) levels.

### 2.5. Statistical Analysis

We analyzed and compared the genotype frequencies of six miRNA polymorphisms between ischemic stroke patients and control subjects using Fisher’s exact test and logistic regression analysis. The associations between genotype frequencies and ischemic stroke were assessed by calculating adjusted odds ratios (AORs) with 95% confidence intervals (CIs), adjusting for age, sex, hypertension, diabetes mellitus, hyperlipidemia, and smoking. Allele combination analysis was also performed to evaluate the association of specific polymorphisms using odds ratios (ORs) and 95% CIs. All polymorphisms were confirmed to be in Hardy–Weinberg equilibrium (HWE, *p* > 0.05). Statistical analyses were conducted using GraphPad Prism version 4.0 (GraphPad Software, Inc., San Diego, CA, USA) and MedCalc version 12.7.7 (MedCalc Software, Mariakerke, Belgium). Haplotype estimations for multiple loci were performed using the expectation-maximization algorithm implemented in HAPSTAT version 3.0 (University of North Carolina, Chapel Hill, NC, USA). To identify polymorphism combinations with potential synergistic effects, all possible combinations were evaluated using the open-source Multifactor Dimensionality Reduction (MDR) software package version 2.0, available at www.epistasis.org (accessed on 30 September 2024). Survival analysis was conducted using the Cox proportional hazards regression model, and statistical significance between groups was determined using the log-rank test. To control the false positive rate in the multiple comparison test, false discovery rate (FDR) correction was performed using the Benjamini–Hochberg method, and the false positive rate was set to not more than 5% [66].

### 2.6. Cell Culture

The human umbilical vein cell line (EA. hy926) was established by fusing primary human umbilical vein cells with a thioguanine-resistant clone of A549 by exposure to polyethylene glycol (PEG). We have cultured the EA. hy926 cells (ATCC, Manassas, VA, USA) using DMEM (Gibco, Grand Island, NY, USA) supplemented with 10% fetal bovine serum (Gibco, Grand Island, NY, USA), 100 U/mL penicillin (Gibco, Grand Island, NY, USA), and 100 μg/mL streptomycin (Gibco, Grand Island, NY, USA). Also, we have cultured the EA. hy926 cells in humidified air with 5% CO_2_ at 37 °C.

### 2.7. Plasmid Amplification, Transformation and Purification

pcDNA^TM^3.1^(−)^ vector was purchased from Thermo Fisher Scientific (Thermo Fisher Scientific, Waltham, MA, USA). The *miR-21* expression vector contained the Precursor-*miR-21* sequence, including *miR-21* rs1292037 T > C and rs13137 A > T in pcDNA^TM^3.1^(−)^. The four *miR-21* expression vectors had haplotypes of *miR-21* rs1292037 T > C and rs13137 A > T polymorphisms as T-A, T-T, C-A, and C-T, respectively. The vector and inserts were ligated at 16 °C for 16 h at a molar ratio of 1:3 using T4 DNA ligase. After the ligation process, this mixture was combined with *E. coli* competent cells DH-5α strain to construct the expression vector. These processes were performed: 10 µL of ligation mixture and 100 uL of competent cells mixed and incubated on ice for 30 min, followed by heat shock at 42 °C for 90 s and incubation on ice for 3 min. We have incubated for 60 min at 37 °C to restore the cells without shaking in 700 µL of Luria–Bertani (LB) broth (BD, Becton, Dickinson and Company, Sparks, MD, USA). Then, the suspended *E. coli* cells in 200 uL of LB broth were spread on the LB-agar plate containing 100 µg/mL of ampicillin and incubated at 37 °C overnight to select the transformants. After overnight incubation, one colony of the plate was transferred to 5 mL of LB broth supplemented with ampicillin (50 µg/mL) for 6 h of pre-incubation at 37 °C before transfer to 200 mL LB broth for a further overnight incubation in the shaking incubator. Furthermore, the *E. coli* was harvested using a centrifuge at 4000 rpm for 10 min, and used as a pellet to extract plasmid DNA using QIAGEN^®^ plasmid midi (Qiagen, Germantown, MD, USA) and AccuPrep^®^ Plasmid Mini Extraction Kit (Bioneer, Daejeon, Republic of Korea).

### 2.8. Over-Expression of the miR-21 Gene in EA. Hy926 Cells

The *miR-21* gene plasmid, including rs1292037 T > C and rs13137 A > T, was used in the transfection process using lipofectamine™ 3000 (Invitrogen, Carlsbad, CA, USA). Furthermore, a combined 3.75 µL of lipofectamine™ 3000 and 5 µg of plasmid DNA were transfected using lipofectamine™ 3000 (Invitrogen, Carlsbad, CA, USA) after 20 h of subculture. After over 16 h of incubation, we washed with Phosphate-Buffered Saline (PBS; Thermo Fisher Scientific, Waltham, MA, USA) and lysed using Trizol and chloroform for RNA extraction.

### 2.9. Quantitative Real-Time PCR

After being transfected for 16 h, the HUVECs cell total RNA was processed using TRIzol (Invitrogen, Carlsbad, CA, USA). Reverse transcription-PCR was performed using an HB_I RT Reaction kit (HeimBiotek, Co., Ltd., Seongnam, Republic of Korea). Quantitative real-time PCR (qPCR) in miRNA detection was performed in a 20 µL reaction containing an HB_I Real-time PCR Master mix kit (HeimBiotek, Co., Ltd., Seongnam, Republic of Korea). Quantitative real-time PCR (qPCR) in target gene detection was performed in a 20 µL reaction containing each sequence-specific primer and quantitative PCR master mix (Solgent, Co., Ltd., Daejeon, Korea). The qPCRs were performed on the Rotor-Gene 6000 real-time PCR system (Qiagen, Co., Ltd., Hilden, Germany). The expression levels were calculated using the comparative threshold cycle (Ct) method and the formula 2-ΔΔCt method.

### 2.10. The Prediction of miR-21 Binding Target Genes

We have confirmed the miRNA sequence using a comprehensive database of miRBase, RNAcentral, and miRPathDB v2.0. Also, the miRNA sequence was confirmed by the National Center for Biotechnology Information (NCBI) (www.ncbi.nlm.nih.gov/, accessed on 30 September 2024). After confirming the miRNA sequence, we used the databases in TargetScan, miRDB, and miRPathDB v2.0 to predict the target genes that bind to *miR-21*. Furthermore, their target gene sequences were also confirmed by the National Center for Biotechnology Information (NCBI) (www.ncbi.nlm.nih.gov/, accessed on 30 September 2024). The prediction between miRNA and target gene binding structure and minimum free energy (MFE) is calculated by the RNAhibrid tool (https://bibiserv.cebitec.uni-bielefeld.de/rnahybrid/, accessed on 30 September 2024). The sequence of pri-*miR-21* confirmed the binding of several target genes, including *RECK*, *FASLG*, *KRIT1*, and *SPRY1*, in prediction databases.

## 3. Results

### 3.1. Clinical Profiles in This Study

Table 1 presents the demographic characteristics and compares clinical variables between ischemic stroke patients and controls. The ischemic stroke group exhibited significant increases in hypertension, diabetes mellitus, smoking, hyperlipidemia, homocysteine levels, and fasting blood sugar, along with a significant decrease in folate levels (*p* < 0.05). The proportions of men in the ischemic stroke and control groups were 41.8% and 46.3%, respectively. The mean ages of patients and controls were 62.48 ± 11.31 years and 62.91 ± 11.28 years, respectively.

### 3.2. Genotype Frequency Analyses of miRNA Polymorphisms in Ischemic Stroke Patients and Controls

We conducted genotype frequency analyses of miRNA polymorphisms in ischemic stroke patients and controls, focusing on *miR-21* rs1292037 T > C, rs13137A > T, *miR-26a* rs7372209 C > T, *miR-124-1* rs531564 G > C, and *miR-126* rs4636297 G > A (Table 2). Adjusted odds ratios (AORs) were calculated via logistic regression analysis. Genotype distributions for the six miRNA polymorphisms conformed to Hardy–Weinberg equilibrium (*p* > 0.05). Significant associations were evident between specific miRNA polymorphisms and ischemic stroke. The *miR-21* rs13137 A > T and *miR-126* rs4636297 G > A polymorphisms displayed significant associations with ischemic stroke prevalence, whereas other miRNA polymorphisms exhibited non-significant trends. The TT genotype of *miR-21* rs13137 was associated with a decreased risk of ischemic stroke (AOR: 0.611, 95% confidence interval [CI]: 0.405–0.923, *p* = 0.019). Although the recessive model (AA + AT vs. TT) showed a trend suggestive of decreased risk (AOR: 0.724, 95% CI: 0.516–1.015, *p* = 0.061), the association was not statistically significant. In contrast, the GA genotype of *miR-126* rs4636297 was associated with an increased risk of ischemic stroke (AOR: 1.557, 95% CI: 1.135–2.135, *p* = 0.006); the dominant model (GG vs. GA + AA) also showed a significant association (AOR: 1.525, 95% CI: 1.123–2.070, *p* = 0.007).

### 3.3. Genotype Frequency Analyses of miRNA Polymorhpisms in Ischemic Stroke Subgroup Patients and Controls

After genotype analysis of the six miRNA polymorphisms had been completed, we assessed whether each polymorphism affected three ischemic stroke subgroups: large artery disease (LAD), small vessel disease (SVD), and cardio-embolism (CE) (Table 3). Subgroup analyses identified associations between the six miRNA polymorphisms and ischemic stroke subtypes. Among subgroups, the *miR-126* rs4636297 G > A polymorphism showed a significant association with ischemic stroke risk in the LAD subgroup (GA genotype, AOR: 1.529, 95% CI: 1.016–2.300, *p* = 0.042). Other miRNA polymorphisms did not show statistically significant associations, although trends were evident. In the SVD subgroup, we observed significant associations with the *miR-21* rs13137 A > T polymorphism (TT genotype, AOR: 0.359, 95% CI: 0.183–0.705, *p* = 0.003; recessive model AA + AT vs. TT, AOR: 0.514, 95% CI: 0.299–0.884, *p* = 0.016) and the *miR-126* rs4636297 G > A polymorphism (GA genotype, AOR: 1.677, 95% CI: 1.075–2.616, *p* = 0.023; dominant model GG vs. GA + AA, AOR: 1.623, 95% CI: 1.054–2.501, *p* = 0.028). Finally, in the CE subgroup, the *miR-21* rs13137 A > T polymorphism was significantly associated with ischemic stroke risk (AT genotype, AOR: 0.432, 95% CI: 0.223–0.837, *p* = 0.013; dominant model AA vs. AT + TT, AOR: 0.476, 95% CI: 0.263–0.861, *p* = 0.014).

### 3.4. Allele Combination Analyses for Each miRNA Plymorphism in Ischemic Stroke Patients and Controls

We next performed allele combination analyses between ischemic stroke patients and controls (Table 4). Several allele combinations showed significant associations with stroke risk (*p* < 0.05), calculated using the multifactor dimensionality reduction method. The A-T-A-A, T-C-A-G, and T-T-G-G allele combinations of *miR-21* rs13137 A > T, *miR-26a* rs7372209C > T, *miR-107* rs2296616 A > G, and *miR-126* rs4636297 G > A were associated with a decreased prevalence of ischemic stroke (OR: 0.479, 95% CI: 0.250–0.917; OR: 0.611, 95% CI: 0.477–0.782; OR: 0.288, 95% CI: 0.123–0.674, respectively). Additionally, the T-T-G and C-T-G allele combinations of *miR-21* rs1292037 T > C, *miR-21* rs13137 A > T, and *miR-126* rs4636297 G > A (OR: 0.155, 95% CI: 0.044–0.544; OR: 0.777, 95% CI: 0.631–0.957) were associated with a decreased prevalence of ischemic stroke. Similarly, the T-T allele combination of *miR-21* rs1292037 T > C and *miR-21* rs13137 A > T (OR: 0.186, 95% CI: 0.062–0.561) and the T-G allele combination of *miR-21* rs13137 A > T and *miR-126* rs4636297 G > A (OR: 0.718, 95% CI: 0.587–0.879) significantly reduced ischemic stroke risk.

### 3.5. Genotype Combination Analyses for Each miRNA Polymorphism in Ischemic Stroke Patients and Controls

We conducted genotype combination analyses for miRNA polymorphisms in ischemic stroke patients and controls (Table 5). The analyses revealed significant associations with ischemic stroke risk (*p* < 0.05); combinations with *p*-values greater than 0.1 were excluded. The TC/TT genotype combination of *miR-21* rs1292037 T > C and *miR-21* rs13137A > T, as well as the TT/GG genotype combination of *miR-21* rs13137 A > T and *miR-126* rs4636297 G > A, were associated with a decreased prevalence of ischemic stroke (TC/TT, AOR: 0.121, *p* = 0.022; TT/GG, AOR: 0.452, *p* = 0.001). Several other genotype combinations involving the *miR-21* rs13137 TT genotype also showed a significant association with decreased ischemic stroke risk. Intriguingly, the TC/AA genotype combination of *miR-21* rs1292037 T > C and *miR-21* rs13137 A > T was associated with an increased prevalence of ischemic stroke. However, when the genotype combination was modified to TC/TT, the risk of ischemic stroke decreased. Finally, various genotype combinations containing the *miR-126* rs4636297 A allele were associated with an increased prevalence of ischemic stroke.

### 3.6. Stratified Analyses of miRNA Genotypes Combined with Clinical Variables Related to Ischemic Stroke

We conducted stratified analyses of the six miRNA genotypes combined with clinical variables related to ischemic stroke (Table S5). These analyses confirmed various combined effects between specific genotypes and ischemic stroke risk factors. The *miR-21* rs1292037 recessive model (TT + TC vs. CC) was associated with an increased prevalence of ischemic stroke among individuals with higher fibrinogen levels (AOR: 6.504, 95% CI: 1.189–35.566); it was associated with a decreased prevalence among individuals with lower fibrinogen levels (AOR: 0.629, 95% CI: 0.396–0.999). In the *miR-26a* rs7372209 recessive model (CC + CT vs. TT), the prevalence of ischemic stroke was higher among individuals older than 63 years (AOR: 2.361, 95% CI: 1.163–4.791); it was lower among individuals younger than 63 years (AOR: 0.474, 95% CI: 0.207–1.084). This polymorphism also significantly increased the prevalence of ischemic stroke in individuals with lower activated partial thromboplastin time (AOR: 16.309, 95% CI: 1.806–147.259); no significant association was observed in those with higher activated partial thromboplastin time (AOR: 0.862, 95% CI: 0.466–1.594). Furthermore, the *miR-126* rs4636297 dominant model (GG vs. GA + AA) was associated with an increased prevalence of ischemic stroke across various risk factor classifications, including higher d-dimer levels (AOR: 18.630, 95% CI: 2.729–127.168). In contrast, the *miR-21* rs13137 recessive model (AA + AT vs. TT) demonstrated a consistent association with decreased ischemic stroke prevalence across clinical risk factors, including hypertension (AOR: 0.539, 95% CI: 0.345–0.841) and lower folate levels (AOR: 0.155, 95% CI: 0.042–0.569).

### 3.7. Ischemic Stroke Prevalence According to Analyses of Interactions Between miRNA Polymorphisms and Environmental Factors

After stratified analyses of miRNA polymorphisms had been completed, we performed interaction analyses between these polymorphisms and various environmental factors, including age, sex, hypertension, diabetes mellitus, hyperlipidemia, and smoking (Table S6). The *miR-21* rs1292037 TC + CC genotype exhibited a synergistic effect, reducing the risk of ischemic stroke in individuals with lower folate levels and higher fasting blood sugar levels. The *miR-21* rs1292037 TC + CC genotype was significantly associated with a decreased risk of ischemic stroke among individuals who exhibited hypertension (AOR: 4.005, 95% CI: 2.491–6.438). Additionally, the *miR-21* rs13137 AT + TT genotype demonstrated a synergistic effect, further decreasing the risk of ischemic stroke in individuals with hypertension and elevated fasting blood sugar levels. Moreover, the combination of *miR-21* rs13137 AT + TT genotype and lower folate levels was associated with a significant reduction in ischemic stroke risk (AOR: 1.496, 95% CI: 1.496–5.068). In contrast, the *miR-26a* rs7372209 CT + TT genotype exhibited a synergistic effect that increased the risk of ischemic stroke in individuals with diabetes mellitus, lower high-density lipoprotein levels, higher low-density lipoprotein levels, elevated fasting blood sugar levels, and higher blood urea nitrogen levels. Similarly, the *miR-124-1* rs531564 GC + CC genotype demonstrated a synergistic effect that increased ischemic stroke risk in the presence of hypertension, smoking, and elevated fasting blood sugar levels. When combined with lower folate levels, the *miR-124-1* rs531564 GC + CC genotype also showed a significant increase in ischemic stroke risk (AOR: 5.580, 95% CI: 2.230–13.961). For the *miR-126* rs4636297 GA + AA genotype, significant synergistic effects were observed in individuals with hypertension (AOR: 4.456, 95% CI: 2.746–7.233) and diabetes mellitus (AOR: 3.705, 95% CI: 1.928–7.118).

### 3.8. Stratified Assessments of Clinical Variables and miRNA Polymorphisms in Ischemic Stroke Patients and Controls According to Analysis of Variance (ANOVA)

In addition to stratifying and interaction analyses, we evaluated associations between miRNA polymorphisms and clinical variables using ANOVA. The *miR-107* rs2296616 A > G genotypes showed a significant association with total cholesterol levels (AA vs. AG vs. GG: AA, 190.68 ± 38.91; AG, 189.63 ± 39.15; GG, 222.82 ± 52.94; *p* = 0.024) (Table 6). Although the *miR-107* rs2296616 A > G genotypes did not demonstrate statistically significant associations with low-density lipoprotein levels or activated partial thromboplastin time, a trend was observed. Similarly, the *miR-21* rs13137 A > T and *miR-26a* rs7372209 C > T genotypes, when combined with folate levels, did not show statistically significant associations but displayed tendencies toward potential relevance.

### 3.9. Comparisons of miRNA Polymorphism Genotype Frequency and Survival in Ischemic Stroke Patients

**Figure 1:**
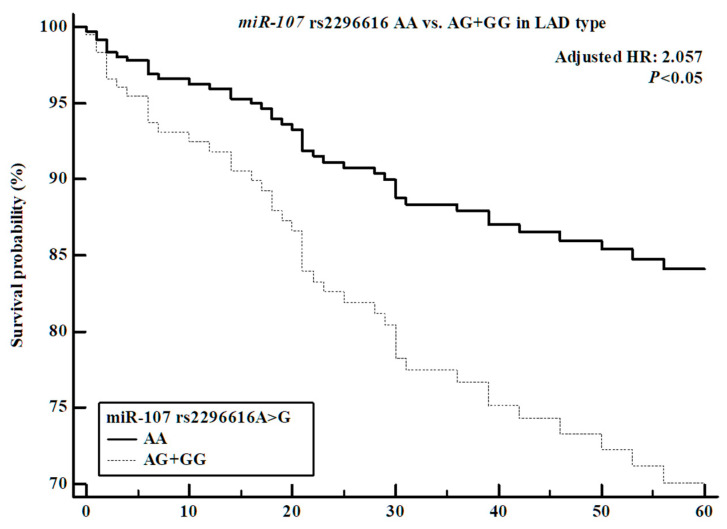
Survival analysis of the *miR-107* rs2296616 dominant model (AA vs. AG + GG) in LAD type of ischemic stroke patients. The *miR-107* rs2296616 AG + GG genotype has confirmed the increasing hazard ratio of ischemic stroke patients (adjusted HR: 2.057, 95% CI: 1.029–4.112, *p* < 0.05).

**Figure 2:**
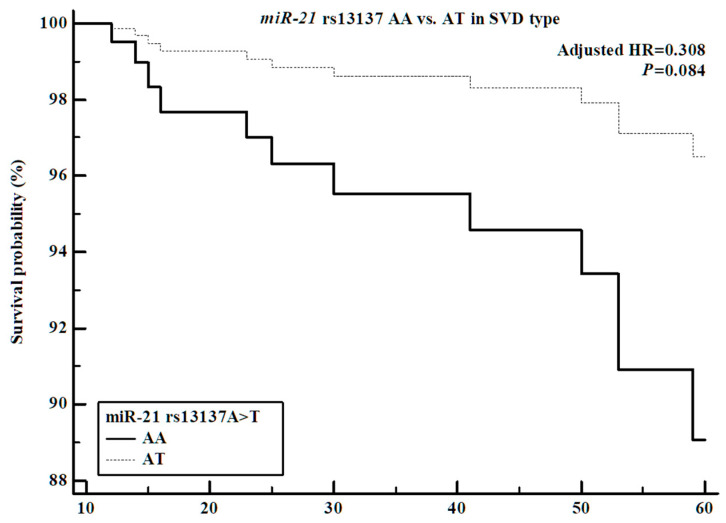
Survival analysis of the *miR-21* rs13137 AT genotype in SVD-type ischemic stroke patients. The *miR-21* rs13137 AT genotype has confirmed the decreasing hazard ratio of ischemic stroke patients (adjusted HR: 0.308, 95% CI: 0.081–1.165, *p* = 0.084), but it did not show a significant *p*-value.

After the ANOVA testing, we performed survival analysis to assess the relationships of six miRNA polymorphisms with survival in ischemic stroke patients. Cox proportional hazards analysis indicated that the miRNA polymorphisms (*miR-21* rs1292037 T > C, rs13137 A > T, *miR-26a* rs7372209 C > T, *miR-107* rs2296616 A > G, *miR-124-1* rs531564 G > C, *miR-126* rs4636297 G > A) were not associated with survival in the overall ischemic stroke cohort. However, significant associations were identified in subgroup analyses. In the Cox proportional analysis of patients in the LAD subgroup, the *miR-107* rs2296616 AG genotype and the dominant model (AA vs. AG + GG) showed significant associations with decreased survival probability (Table S7, Figure 1). Additionally, the *miR-21* rs13137 AT genotype exhibited a trend toward increased survival probability, although the association was not statistically significant (Table S8, Figure 2). Finally, Cox proportional analysis in the CE subgroup revealed a significant association between the *miR-26a* rs7372209 TT genotype and survival (Table S9).

### 3.10. Differences in miR-21 Expression Between miR-21 rs1292037 and rs13137 Polymorphisms

**Figure 3:**
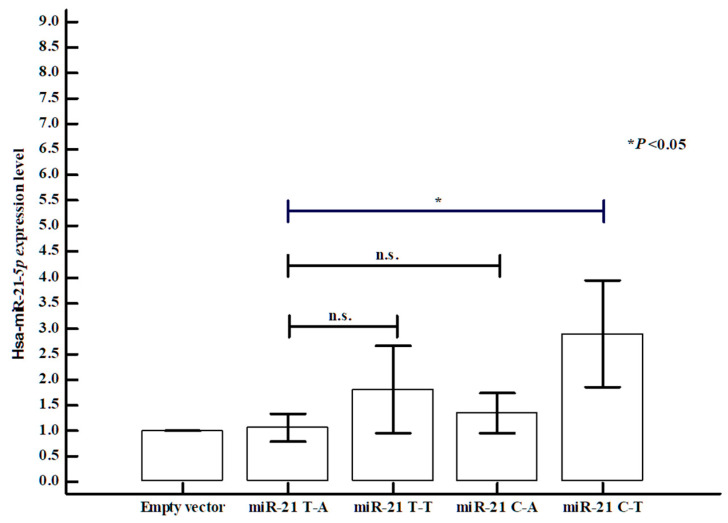
The difference of *miR-21* mRNA expression levels between respective genotypes of *miR-21* rs1292037 T > C and rs13137 A > T polymorphisms in HUVECs (EA.hy926 cells). The different *miR-21* mRNA expression levels show that individual polymorphism combinations could affect their expression. The bars showed the mean ± SD from cell lines. The values are normalized to RNU6B mRNA levels. RNU6B, RNA, U6 small nuclear 6, pseudogene.

**Figure 4:**
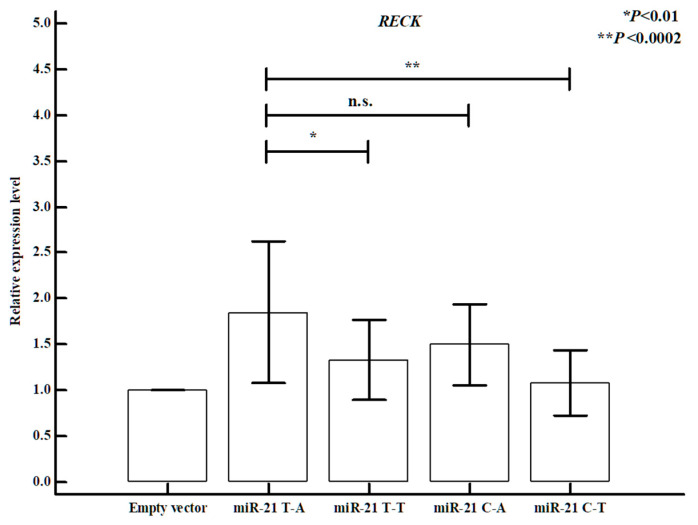
The difference of *RECK* mRNA expression levels between respective genotypes of *miR-21* rs1292037T > C and rs13137A > T polymorphisms in HUVECs (EA.hy926 cells). The different *RECK* mRNA expression levels show that individual polymorphism combinations could affect their expression. The bars showed the mean ± SD from cell lines. The values are normalized to GAPDH mRNA levels. GAPDH, glyceraldehyde 3-phosphate dehydrogenase.

Finally, we investigated potential target genes and reviewed reference studies related to *miR-21* binding. Through transfection experiments using a *miR-21* expression vector in EA.hy926 cells, we found that *miR-21* mRNA expression levels differed according to haplotypes formed by *miR-21* rs1292037 and rs13137 polymorphisms (Figure 3). Analysis of *miR-21* expression levels demonstrated that the T-A haplotype (rs1292037-rs13137) resulted in significantly lower expression compared with the C-T haplotype. Moreover, expression levels of predicted target genes, such as *RECK*, significantly differed according to *miR-21* rs1292037-rs13137 haplotype (Figure 4).

## 4. Discussion

This study aimed to identify novel markers that could potentially be used for clinical diagnosis. We selected six single-nucleotide polymorphisms—*miR-21* rs1292037 T > C, rs13137 A > T, *miR-26a* rs7372209 C > T, *miR-107* rs2296616 A > G, *miR-124-1* rs531564 G > C, and *miR-126* rs4636297 G > A—based on their potential functional relevance. We then investigated associations of these gene polymorphisms with ischemic stroke risk and compared exosomal miRNA expression levels between ischemic stroke patients and controls. Our results confirmed that *miR-21* rs13137 A > T and *miR-126* rs4636297 G > A were significantly associated with the prevalence of ischemic stroke. Although other miRNA gene polymorphisms did not show statistically significant associations, several demonstrated a tendency to influence ischemic stroke risk. Specific genotypes were related to subtypes of ischemic stroke, including LAD (*miR-126* rs4636297 GA), SVD (*miR-21* rs13137 TT, recessive model; *miR-126* rs4636297 GA, dominant model), and CE (*miR-21* rs13137 AT, dominant model). Moreover, several dominant models of miRNA polymorphisms (*miR-21* rs1292037, rs13137; *miR-26a* rs7372209; *miR-107* rs2296616; *miR-124-1* rs531564; *miR-126* rs4636297) exhibited synergistic effects on ischemic stroke risk. Allele combination and genotype combination analyses also indicated that the T allele of *miR-21* rs13137 was associated with a decreased prevalence of ischemic stroke, whereas the A allele of *miR-126* rs4636297 was associated with an increased prevalence. These findings suggest that several miRNA polymorphisms are associated with ischemic stroke risk.

Previous studies have revealed that *miR-21* plays a role in ischemic stroke by regulating target gene expression and that polymorphisms in the *miR-21* gene are associated with ischemic stroke risk [53,67]. Additionally, *miR-21* has been suggested to constitute a serum diagnostic marker after stroke [68]. Based on this evidence, we hypothesized that *miR-21* polymorphisms influence ischemic stroke prevalence and prognosis by affecting *miR-21* expression levels. Consistent with this hypothesis, we identified a significant association between the *miR-21* rs13137 A > T genotype and decreased ischemic stroke prevalence. Moreover, interaction analysis revealed that the *miR-21* rs13137 A > T dominant model was associated with reduced risks of ischemic stroke among individuals with lower folate levels, hypertension, and elevated fasting blood sugar levels. Lower folate levels and higher homocysteine levels are established risk factors for ischemic stroke [69]; elevated fasting blood sugar levels have been identified as an independent risk factor for ischemic stroke [70]. Notably, associations between *miR-126* gene polymorphisms and ischemic stroke risk have been detected in various non-Korean populations [53]. Furthermore, other studies have shown that the *miR-126* gene influences the brain-heart relationship after ischemic stroke and that *miR-126* expression may serve as a novel marker for ischemic stroke [48,71]. Based on these findings, we hypothesized that *miR-126* could act as a novel diagnostic marker and that its gene polymorphisms might regulate *miR-126* expression. Accordingly, we speculated that *miR-126* polymorphisms could affect ischemic stroke prevalence and prognosis by regulating *miR-126* expression. Our results confirmed that the *miR-126* rs4636297 G > A genotype was significantly associated with an increased prevalence of ischemic stroke. Furthermore, the *miR-126* rs4636297 G > A genotype demonstrated consistent associations with the LAD and SVD subtypes of ischemic stroke. Additionally, the *miR-126* rs4636297 G > A dominant model was associated with an increased prevalence of ischemic stroke among individuals with hypertension, diabetes mellitus, and elevated fasting blood sugar levels. Hypertension and diabetes mellitus are well-established risk factors for ischemic stroke [9,72], and elevated fasting blood sugar levels have been described as an independent risk factor for ischemic stroke [73].

Emerging evidence underscores the pivotal roles of *miR-21* and *miR-126* in regulating angiogenesis and vascular repair following ischemic stroke. *miR-21* not only mitigates neuronal apoptosis and inflammatory responses but also enhances post-ischemic neurovascular remodeling, thereby supporting functional recovery [74,75,76]. Concurrently, *miR-126* is recognized as an essential regulator of endothelial integrity and vascular homeostasis, facilitating vascular remodeling and protecting the cardiovascular system during ischemic injury [71,77]. Both miRNAs have been identified as promising therapeutic targets, with preclinical studies demonstrating that modulation of their expression can improve angiogenic capacity, restore blood flow, and promote neurofunctional recovery after stroke [78,79]. These findings collectively suggest that therapeutic strategies aimed at enhancing or restoring *miR-21* and *miR-126* activity may hold significant potential for improving outcomes in ischemic stroke patients. Based on our findings, we propose that several miRNA gene polymorphisms, particularly *miR-21* and *miR-126*, influence ischemic stroke prevalence and could serve as diagnostic markers for ischemic stroke.

## 5. Conclusions

We confirmed that the *miR-21* rs13137 A > T and *miR-126* rs4636297 G > A polymorphisms are genetically associated with ischemic stroke risk and prognosis. Our findings suggest that miRNA gene polymorphisms influence the diagnosis of ischemic stroke and hold potential as biomarkers for stroke risk prediction. However, this study had some limitations. First, although our results suggest that miRNA polymorphisms can be used to predict phenotypes associated with ischemic stroke prevalence, we have not validated these findings in an in vivo mouse model. Second, the study population exclusively consisted of Korean individuals; our results require validation in other groups, such as Western populations. Due to ethnic variations in stroke subtype and genotype frequencies, our findings may have limited generalizability. If future studies confirm a critical role for miRNA gene polymorphisms in ischemic stroke incidence, approaches that regulate miRNA expression or activity may improve stroke prevention and enhance angiogenic capacity after ischemic injury. Therefore, further research involving heterogeneous cohorts is needed to better understand the influence of miRNA genetic variants. In future work, we aim to validate these findings by analyzing specific expression level differences between ischemic stroke patients and controls via small RNA sequencing methods.

## Figures and Tables

**Table 1:** Comparison of baseline characteristics between ischemic stroke patients and controls.

Characteristics	Controls	Stroke Patients	*p* ^a^
	(n = 400)	(n = 520)	
Males, n (%)	167 (41.8)	241 (46.3)	0.164
Age (years, mean ± SD)	62.48 ± 11.31	62.91 ± 11.28	0.560
Smoking, n (%)	131 (32.8)	208 (40.0)	0.024
Hypertension, n (%)	161 (40.3)	332 (63.8)	<0.0001
Diabetes mellitus, n (%)	55 (13.8)	140 (26.9)	<0.0001
Hyperlipidemia, n (%)	86 (21.5)	157 (30.2)	0.003
HDL-c (mg/dL, mean ± SD)	46.47 ± 14.07	44.51 ± 15.47	0.146
LDL-c (mg/dL, mean ± SD)	116.63 ± 40.64	120.37 ± 34.00	0.059 ^b^
Homocysteine (μmol/L, mean ± SD)	9.99 ± 4.16	11.14 ± 6.54	0.001 ^b^
Folate (nmol/L, mean ± SD)	8.98 ± 8.05	6.83 ± 5.13	<0.0001 ^b^
Vitamin B_12_ (pg/mL, mean ± SD)	746.78 ± 677.94	754.99 ± 652.55	0.854
Fasting blood sugar (mg/dL, mean ± SD)	115.12 ± 38.18	136.33 ± 59.36	<0.0001 ^b^
Total cholesterol (mg/dL, mean ± SD)	192.15 ± 37.45	189.96 ± 40.56	0.406
Triglyceride (mg/dL, mean ± SD)	144.01 ± 84.72	148.99 ± 93.04	0.409
Platelets (10^3^/µL, mean ± SD)	243.63 ± 64.01	245.41 ± 75.57	0.633 ^b^
Prothrombin time (sec, mean ± SD)	11.76 ± 0.78	11.92 ± 3.27	0.565 ^b^
aPTT (sec, mean ± SD)	31.90 ± 8.31	30.53 ± 4.67	0.134 ^b^
Fibrinogen (mg/dL, mean ± SD)	418.21 ± 144.98	426.97 ± 131.02	0.494
Antithrombin (%, mean ± SD)	91.26 ± 17.41	93.71 ± 17.49	0.149
Creatinine (mg/dL, mean ± SD)	0.96 ± 0.25	1.03 ± 0.77	0.571 ^b^
Blood urea nitrogen (mg/dL, mean ± SD)	15.72 ± 5.01	16.33 ± 7.52	0.566 ^b^
Uric acid (mg/dL, mean ± SD)	4.64 ± 1.46	4.69 ± 1.54	0.662

SD, standard deviation; HDL-c, high density lipoprotein cholesterol; LDL-c, low density lipoprotein; aPTT, activated partial thromboplastin time. ^a^ *p*-values were calculated by two-sided *t*-test for continuous variables and chi-square test for categorical variables. ^b^ *p*-values were calculated by Mann–Whitney test for continuous variables.

**Table 2:** Genotype frequency analyses of the six miRNA polymorphisms between ischemic stroke patients and controls.

Genotypes	Controls	Stroke Patients	AOR (95% CI) *	*p* ^†^	FDR-*P*
	(n = 400)	(n = 520)			
*miR-21* rs1292037 T > C					
TT	102 (25.5)	120 (23.1)	1.000 (reference)		
TC	187 (46.8)	268 (51.5)	1.214 (0.863–1.709)	0.265	0.552
CC	111 (27.8)	132 (25.4)	0.938 (0.635–1.387)	0.749	0.899
Dominant (TT vs. TC + CC)			1.111 (0.809–1.527)	0.516	0.658
Recessive (TT + TC vs. CC)			0.837 (0.614–1.143)	0.263	0.526
HWE-*P*	0.197	0.475			
*miR-21* rs13137 A > T					
AA	99 (24.8)	150 (28.8)	1.000 (reference)		
AT	208 (52.0)	270 (51.9)	0.833 (0.599–1.158)	0.276	0.552
TT	93 (23.3)	100 (19.2)	**0.611 (0.405–0.923)**	**0.019**	0.114
Dominant (AA vs. AT + TT)			0.772 (0.566–1.053)	0.103	0.309
Recessive (AA + AT vs. TT)			0.724 (0.516–1.015)	0.061	0.366
HWE-*P*	0.421	0.272			
*miR-26a* rs7372209 C > T					
CC	214 (53.5)	275 (52.9)	1.000 (reference)		
CT	155 (38.8)	203 (39.0)	1.059 (0.793–1.414)	0.698	0.698
TT	31 (7.8)	42 (8.1)	1.259 (0.738–2.146)	0.398	0.796
Dominant (CC vs. CT + TT)			1.088 (0.826–1.434)	0.548	0.658
Recessive (CC + CT vs. TT)			1.212 (0.729–2.015)	0.459	0.689
HWE-*P*	0.691	0.598			
*miR-107* rs2296616 A > G					
AA	329 (82.3)	426 (81.9)	1.000 (reference)		
AG	66 (16.5)	88 (16.9)	1.081 (0.748–1.564)	0.679	0.698
GG	5 (1.3)	6 (1.2)	1.072 (0.309–3.719)	0.913	0.913
Dominant (AA vs. AG + GG)			1.079 (0.755–1.543)	0.677	0.677
Recessive (AA + AG vs. GG)			1.053 (0.304–3.651)	0.935	0.935
HWE-*P*	0.419	0.547			
*miR-124-1* rs531564 G > C					
GG	302 (75.5)	374 (71.9)	1.000 (reference)		
GC	88 (22.0)	129 (24.8)	1.134 (0.817–1.573)	0.454	0.681
CC	10 (2.5)	17 (3.3)	1.760 (0.770–4.024)	0.180	0.540
Dominant (GG vs. GC + CC)			1.191 (0.870–1.628)	0.275	0.550
Recessive (GG + GC vs. CC)			1.711 (0.751–3.898)	0.201	0.526
HWE-*P*	0.246	0.161			
*miR-126* rs4636297 G > A					
GG	296 (74.0)	349 (67.1)	1.000 (reference)		
GA	93 (23.3)	158 (30.4)	**1.557 (1.135–2.135)**	**0.006**	**0.036**
AA	11 (2.8)	13 (2.5)	1.158 (0.486–2.756)	0.741	0.899
Dominant (GG vs. GA + AA)			**1.525 (1.123–2.070)**	**0.007**	**0.042**
Recessive (GG + GA vs. AA)			1.037 (0.437–2.459)	0.934	0.935
HWE-*P*	0.267	0.324			

AOR, adjusted odds ratio; HWE, Hardy–Weinberg equilibrium; 95% CI, 95% confidence interval; FDR, false discovery rate. * Adjusted by age, sex, hypertension, diabetes mellitus, hyperlipidemia, and smoking. ^†^ *p*-value calculated by logistics regression analysis; *p*-values < 0.05 are bold.

**Table 3:** Genotype frequency analyses of the six-miRNA polymorphism between ischemic stroke subgroup patients and controls.

Genotypes	Controls	LAD Patients	AOR (95% CI) *	*p* ^†^	SVD Patients	AOR (95% CI) *	*p* ^†^	CE Patients	AOR (95% CI) *	*p* ^†^
	(n = 400)	(n = 204)			(n = 148)			(n = 58)		
*miR-21* rs1292037 T > C										
TT	102 (25.5)	48 (23.5)	1.000 (reference)		38 (25.7)	1.000 (reference)		19 (32.8)	1.000 (reference)	
TC	187 (46.8)	105 (51.5)	1.174 (0.753–1.829)	0.479	74 (50.0)	0.938 (0.577–1.526)	0.797	25 (43.1)	0.702 (0.361–1.366)	0.298
CC	111 (27.8)	51 (25.0)	0.852 (0.504–1.440)	0.549	36 (24.3)	0.719 (0.404–1.280)	0.262	14 (24.1)	0.653 (0.302–1.415)	0.280
Dominant (TT vs. TC + CC)			1.063 (0.702–1.609)	0.773		0.866 (0.549–1.366)	0.537		0.688 (0.375–1.261)	0.226
Recessive (TT + TC vs. CC)			0.800 (0.531–1.207)	0.288		0.768 (0.482–1.223)	0.265		0.804 (0.419–1.542)	0.511
*miR-21* rs13137 A > T										
AA	99 (24.8)	60 (29.4)	1.000 (reference)		44 (29.7)	1.000 (reference)		23 (39.7)	1.000 (reference)	
AT	208 (52.0)	101 (49.5)	0.776 (0.505–1.193)	0.248	81 (54.7)	0.742 (0.466–1.183)	0.210	22 (37.9)	**0.432** **(0.223–0.837)**	**0.013**
TT	93 (23.3)	43 (21.1)	0.638 (0.373–1.091)	0.100	23 (15.5)	**0.359** **(0.183–0.705)**	**0.003**	13 (22.4)	0.516 (0.237–1.120)	0.094
Dominant (AA vs. AT + TT)			0.745 (0.499–1.114)	0.152		0.640 (0.409–1.002)	0.051		**0.476** **(0.263–0.861)**	**0.014**
Recessive (AA + AT vs. TT)			0.809 (0.523–1.251)	0.341		**0.514** **(0.299–0.884)**	**0.016**		0.900 (0.460–1.761)	0.758
*miR-26a* rs7372209 C > T										
CC	214 (53.5)	110 (53.9)	1.000 (reference)		83 (56.1)	1.000 (reference)		25 (43.1)	1.000 (reference)	
CT	155 (38.8)	76 (37.3)	0.951 (0.649–1.394)	0.796	55 (37.2)	0.932 (0.609–1.426)	0.745	27 (46.6)	1.481 (0.820–2.674)	0.193
TT	31 (7.8)	18 (8.8)	1.491 (0.750–2.966)	0.254	10 (6.8)	1.130 (0.510–2.504)	0.763	6 (10.3)	2.049 (0.735–5.711)	0.170
Dominant (CC vs. CT + TT)			1.025 (0.714–1.471)	0.895		0.961 (0.643–1.438)	0.848		1.566 (0.888–2.761)	0.121
Recessive (CC + CT vs. TT)			1.500 (0.778–2.893)	0.227		1.157 (0.536–2.497)	0.710		1.571 (0.612–4.031)	0.348
*miR-107* rs2296616 A > G										
AA	329 (82.3)	169 (82.8)	1.000 (reference)		119 (80.4)	1.000 (reference)		48 (82.8)	1.000 (reference)	
AG	66 (16.5)	33 (16.2)	1.016 (0.624–1.653)	0.949	28 (18.9)	1.294 (0.769–2.179)	0.332	10 (17.2)	1.111 (0.529–2.336)	0.781
GG	5 (1.3)	2 (1.0)	0.712 (0.126–4.031)	0.701	1 (0.7)	0.575 (0.058–5.757)	0.638	0 (0.0)	-	0.996
Dominant (AA vs. AG + GG)			0.990 (0.617–1.589)	0.966		1.241 (0.745–2.066)	0.407		1.025 (0.490–2.144)	0.948
Recessive (AA + AG vs. GG)			0.688 (0.121–3.905)	0.673		0.558 (0.057–5.438)	0.616		-	0.996
*miR-124-1* rs531564 G > C										
GG	302 (75.5)	147 (72.1)	1.000 (reference)		115 (77.7)	1.000 (reference)		41 (70.7)	1.000 (reference)	
GC	88 (22.0)	49 (24.0)	1.097 (0.715–1.683)	0.671	31 (20.9)	0.793 (0.482–1.306)	0.363	15 (25.9)	1.235 (0.647–2.360)	0.522
CC	10 (2.5)	8 (3.9)	2.168 (0.797–5.893)	0.130	2 (1.4)	0.546 (0.111–2.689)	0.457	2 (3.4)	1.304 (0.259–6.573)	0.748
Dominant (GG vs. GC + CC)			1.196 (0.797–1.795)	0.388		0.776 (0.479–1.257)	0.303		1.258 (0.675–2.341)	0.470
Recessive (GG + GC vs. CC)			2.124 (0.784–5.756)	0.139		0.600 (0.123–2.930)	0.528		1.427 (0.289–7.046)	0.662
*miR-126* rs4636297 G > A										
GG	296 (74.0)	140 (68.6)	1.000 (reference)		96 (64.9)	1.000 (reference)		41 (70.7)	1.000 (reference)	
GA	93 (23.3)	60 (29.4)	**1.529** **(1.016–2.300)**	**0.042**	48 (32.4)	**1.677** **(1.075–2.616)**	**0.023**	14 (24.1)	1.172 (0.603–2.277)	0.641
AA	11 (2.8)	4 (2.0)	0.946 (0.271–3.304)	0.930	4 (2.7)	1.078 (0.316–3.681)	0.904	3 (5.2)	2.781 (0.689–11.217)	0.151
Dominant (GG vs. GA + AA)			1.481 (0.995–2.205)	0.053		**1.623** **(1.054–2.501)**	**0.028**		1.318 (0.706–2.462)	0.386
Recessive (GG + GA vs. AA)			0.879 (0.255–3.033)	0.839		0.959 (0.285–3.228)	0.946		2.771 (0.705–10.886)	0.144

AOR, adjusted odds ratio; 95% CI, 95% confidence interval; LAD, large artery disease; SVD, small vessel disease; CE, cardio-embolism. * Adjusted by age, sex, hypertension, diabetes mellitus, hyperlipidemia, and smoking. ^†^ *p*-value calculated by logistics regression analysis; *p*-values < 0.05 are bold.

**Table 4:** Allele combination analyses for the miRNA polymorphisms in ischemic stroke patients and controls.

Allele Combinations	Controls	Stroke	OR (95% CI)	*p*
	(2n = 800)	(2n = 1040)		
*miR-21* rs13137 A > T/*miR-26a* rs7372209 C > T/*miR-107* rs2296616 A > G/*miR-126* rs4636297 G > A *				
A-C-A-G	219 (27.4)	342 (32.9)	1.000 (reference)	
A-C-A-A	37 (4.7)	43 (4.2)	0.753 (0.470–1.206)	0.244
A-C-G-G	27 (3.4)	26 (2.5)	0.624 (0.355–1.098)	0.105
A-C-G-A	5 (0.6)	12 (1.1)	1.555 (0.541–4.476)	0.378
A-T-A-G	86 (10.8)	114 (10.9)	0.859 (0.619–1.192)	0.365
A-T-A-A	23 (2.9)	17 (1.6)	**0.479 (0.250–0.917)**	**0.025**
A-T-G-G	6 (0.8)	15 (1.5)	1.620 (0.619–4.239)	0.285
A-T-G-A	2 (0.3)	1 (0.1)	0.324 (0.029–3.595)	0.316
T-C-A-G	242 (30.2)	228 (21.9)	**0.611 (0.477–0.782)**	**<0.0001**
T-C-A-A	35 (4.4)	74 (7.1)	1.370 (0.886–2.120)	0.143
T-C-G-G	16 (2.0)	29 (2.8)	1.175 (0.623–2.213)	0.612
T-C-G-A	1 (0.2)	0 (0.0)	-	-
T-T-A-G	70 (8.8)	94 (9.1)	0.870 (0.612–1.238)	0.443
T-T-A-A	11 (1.4)	29 (2.8)	1.709 (0.836–3.491)	0.108
T-T-G-G	18 (2.3)	8 (0.8)	**0.288 (0.123–0.674)**	**0.001**
T-T-G-A	0 (0.0)	9 (0.9)	-	-
*miR-21* rs1292037 T > C/*miR-21* rs13137 A > T/*miR-126* rs4636297 G > A *				
T-A-G	316 (39.5)	437 (42.0)	1.000 (reference)	
T-A-A	59 (7.4)	66 (6.3)	0.809 (0.553–1.183)	0.277
T-T-G	14 (1.7)	3 (0.3)	**0.155 (0.044–0.544)**	**<0.0001**
T-T-A	2 (0.3)	2 (0.2)	0.723 (0.101–5.161)	0.749
C-A-G	21 (2.6)	56 (5.4)	**1.928 (1.144–3.250)**	**0.006**
C-A-A	10 (1.2)	10 (1.0)	0.723 (0.297–1.758)	0.478
C-T-G	334 (41.8)	359 (34.5)	**0.777 (0.631–0.957)**	**0.017**
C-T-A	44 (5.5)	107 (10.3)	**1.758 (1.203–2.571)**	**0.002**
*miR-21* rs1292037 T > C/*miR-21* rs13137 A > T				
T-A	375 (46.9)	504 (48.4)	1.000 (reference)	
T-T	16 (2.0)	4 (0.4)	**0.186 (0.062–0.561)**	**<0.0001**
C-A	31 (3.8)	66 (6.4)	**1.584 (1.013–2.477)**	**0.033**
C-T	378 (47.3)	466 (44.8)	0.917 (0.758–1.110)	0.374
*miR-21* rs13137 A > T/*miR-126* rs4636297 G > A *				
A-G	339 (42.4)	494 (47.5)	1.000 (reference)	
A-A	67 (8.4)	76 (7.3)	0.778 (0.545–1.112)	0.172
T-G	346 (43.2)	362 (34.8)	**0.718 (0.587–0.879)**	**0.001**
T-A	48 (6.0)	108 (10.4)	**1.544 (1.070–2.229)**	**0.015**

OR, odds ratio; 95% CI, 95% confidence interval. *p*-values < 0.05 are bold. * The allele combination types were calculated by MDR method.

**Table 5:** Genotype combination analyses for the miRNA polymorphisms in ischemic stroke patients and controls.

Genotype Combinations	Controls	Stroke	AOR (95% CI)	*p* *
	(n = 400)	(n = 520)		
*miR-21* rs1292037 T > C/*miR-21* rs13137 A > T				
TT/AA	95 (23.8)	118 (22.7)	1.000 (reference)	
TC/AA	4 (1.0)	25 (4.8)	**10.549 (3.141–35.427)**	**0.0001**
TC/TT	7 (1.8)	2 (0.4)	**0.121 (0.020–0.733)**	**0.022**
*miR-21* rs1292037 T > C/*miR-126* rs4636297 G > A				
TT/GG	76 (19.0)	93 (17.9)	1.000 (reference)	
TC/GA	43 (10.8)	90 (17.3)	**1.903 (1.137–3.186)**	**0.014**
*miR-21* rs13137 A > T/*miR-26a* rs7372209 C > T				
AA/CC	47 (11.8)	80 (15.4)	1.000 (reference)	
TT/CC	53 (13.3)	47 (9.0)	**0.443 (0.244–0.807)**	**0.008**
*miR-21* rs13137 A > T/*miR-107* rs2296616 A > G				
AA/AA	82 (20.5)	122 (23.5)	1.000 (reference)	
TT/AA	81 (20.3)	82 (15.8)	**0.575 (0.363–0.910)**	**0.018**
*miR-21* rs13137 A > T/*miR-126* rs4636297 G > A				
AA/GG	75 (18.8)	116 (22.3)	1.000 (reference)	
TT/GG	77 (19.3)	64 (12.3)	**0.452 (0.280–0.731)**	**0.001**
*miR-26a* rs7372209 C > T/*miR-126* rs4636297 G > A				
CC/GG	163 (40.8)	187 (36.0)	1.000 (reference)	
CC/GA	44 (11.0)	80 (15.4)	**1.888 (1.196–2.981)**	**0.006**
*miR-107* rs2296616 A > G/*miR-124-1* rs531564 G > C				
AA/GG	244 (61.0)	309 (59.4)	1.000 (reference)	
AG/GC	9 (2.3)	25 (4.8)	**2.463 (1.099–5.519)**	**0.029**
GG/CC	0 (0.0)	0 (0.0)	-	-
*miR-107* rs2296616 A > G/*miR-126* rs4636297 G > A				
AA/GG	242 (60.5)	288 (55.4)	1.000 (reference)	
AA/GA	77 (19.3)	130 (25.0)	**1.580 (1.114–2.242)**	**0.010**
GG/AA	0 (0.0)	0 (0.0)	-	-
*miR-124-1* rs531564 G > C/*miR-126* rs4636297 G > A				
GG/GG	226 (56.5)	244 (46.9)	1.000 (reference)	
GG/GA	66 (16.5)	119 (22.9)	**1.843 (1.272–2.672)**	**0.001**

AOR, adjusted odds ratio; 95% CI, 95% confidence interval. * Adjusted by age, sex, hypertension, diabetes mellitus, hyperlipidemia, and smoking. *p*-values < 0.05 are bold and >0.1 are exclude.

**Table 6:** Clinical factors stratify analyses of ischemic stroke patients and control groups according to the six miRNA polymorphisms using analysis of variance (ANOVA).

Genotypes	LDL-Cholsterol (mg/dL)		Folate (mg/mL)		Total Cholesterol (mg/dL)		aPTT (sec)		Fibrinogen (mg/dL)	
	Mean ± SD	*p* *	Mean ± SD	*p* *	Mean ± SD	*p* *	Mean ± SD	*p* *	Mean ± SD	*p* *
*miR-21* rs1292037 T > C										
TT	118.18 ± 37.89	0.753	7.37 ± 5.30	0.575	189.64 ± 41.79	0.798	30.98 ± 7.22	0.709	409.55 ± 137.03	0.157
TC	120.47 ± 36.34		7.94 ± 6.53		190.88 ± 37.14		30.87 ± 5.40		434.50 ± 134.37	
CC	118.56 ± 32.92		7.77 ± 7.83		192.11 ± 40.76		31.31 ± 6.74		420.38 ± 130.83	
*miR-21* rs13137 A > T										
AA	121.71 ± 38.10	0.593	7.09 ± 4.83	0.086	192.51 ± 42.70	0.605	30.87 ± 6.94	0.911	409.23 ± 128.11	0.130
AT	118.35 ± 35.56		7.81 ± 6.32		189.65 ± 36.94		31.10 ± 5.87		434.33 ± 134.73	
TT	119.19 ± 33.30		8.49 ± 8.96		191.89 ± 40.21		30.98 ± 6.20		420.83 ± 140.53	
*miR-26a* rs7372209 C > T										
CC	119.64 ± 35.11	0.890	7.95 ± 7.79	0.096	190.56 ± 38.16	0.696	30.89 ± 6.01	0.820	421.94 ± 127.56	0.821
CT	119.54 ± 37.97		7.25 ± 4.91		190.59 ± 40.91		31.19 ± 6.52		428.18 ± 141.55	
TT	116.96 ± 29.18		8.91 ± 5.37		194.68 ± 38.55		30.96 ± 6.35		430.93 ± 147.15	
*miR-107* rs2296616 A > G										
AA	119.14 ± 36.22	0.062	7.86 ± 6.99	0.316	**190.68 ± 38.91**	**0.024**	31.05 ± 6.21	0.093	426.79 ± 132.88	0.759
AG	118.76 ± 31.09		7.12 ± 4.74		**189.63 ± 39.15**		31.19 ± 6.45		416.12 ± 144.11	
GG	151.14 ± 58.25		9.42 ± 4.37		**222.82 ± 52.94**		26.97 ± 3.69		425.03 ± 64.79	
*miR-124-1* rs531564 G > C										
GG	120.68 ± 36.30	0.304	7.73 ± 6.73	0.945	191.83 ± 39.58	0.397	31.02 ± 6.51	0.997	422.70 ± 127.94	0.642
GC	116.50 ± 34.49		7.78 ± 6.38		189.00 ± 38.80		30.99 ± 5.58		428.54 ± 151.78	
CC	112.65 ± 34.06		8.16 ± 6.58		183.44 ± 34.55		31.09 ± 4.46		449.24 ± 140.30	
*miR-126* rs4636297 G > A										
GG	119.37 ± 37.71	0.972	7.91 ± 6.97	0.554	191.56 ± 40.74	0.383	30.82 ± 6.25	0.176	424.51 ± 131.80	0.955
GA	119.37 ± 30.70		7.37 ± 5.84		188.50 ± 34.89		31.30 ± 6.05		425.20 ± 142.83	
AA	121.77 ± 37.50		7.80 ± 5.38		198.35 ± 41.33		33.48 ± 7.89		435.95 ± 92.08	

ANOVA, analysis of variance; LDL-c, low density lipoprotein cholesterol; aPTT, activated partial thromboplastin time; SD, standard deviation. * Calculated using ANOVA. *p*-values < 0.05 are bold.

## Data Availability

The data presented in this study can be made available upon request from the corresponding author.

## Supplementary Materials

The following supporting information can be downloaded at: https://www.mdpi.com/article/10.3390/cells14171389/s1, Table S1: Information of *miR-21* polymorphisms for population diversity frequency; Table S2: Information of *miR-26a* polymorphism for population diversity frequency; Table S3: Information of *miR-107* and 124-1 polymorphism for population diversity frequency; Table S4: Information of *miR-126* polymorphism for population diversity frequency; Table S5: Stratified analysis between the six miRNA genotypes and clinical factors of ischemic stroke; Table S6: Ischemic stroke prevalence analyses between the six miRNA polymorphisms and several environmental factors using interaction analysis; Table S7: Comparison of the six miRNA polymorphism frequencies for ischemic stroke LAD patient’s survival; Table S8: Comparison of the six miRNA polymorphism frequencies for ischemic stroke SVD patient’s survival. Table S9: Comparison of the six miRNA polymorphism frequencies for ischemic stroke CE patients′ survival.

## Abbreviations

The following abbreviations are used in this manuscript:

MiRNA	MicroRNA
Dicer-1	Dicer 1, ribonuclease III
STAT3	Signal transducer and activator of transcription 3
TOAST	Trial of Org 10172 in Acute Stroke Treatment
LAD	Large-artery disease
MRI	Magnetic resonance imaging
SVD	Small-vessel disease
CE	Cardio-embolism
HTN	Hypertension
DM	Diabetes mellitus
PCR	Polymerase chain reaction
RFLP	Restriction fragment length polymorphism
SNP	Single-nucleotide polymorphism
tHcy	Total homocysteine
FPIA	Fluorescent polarizing immunoassay
HDL-C	High-density lipoprotein-cholesterol
AOR	Adjusted odds ratios
CI	Confidence intervals
OR	Odds ratios
HWE	Hardy–Weinberg equilibrium
MDR	Multifactor Dimensionality Reduction
ANOVA	Analysis of variance
RECK	Reversion-inducing cysteine-rich protein with Kazal motifs
HUVECs	Human Umbilical Vein Endothelial Cells
