# Supplementary material for: Prognostic Associations and Functional Implications of Angiogenesis-Related miRNA Variants in Ischemic Stroke
Source: Cells. 2025 Sep 5;14(17):1389. doi: 10.3390/cells14171389 (PMC12428635; doi:10.3390/cells14171389)
Supplement: Supplementary file 1 [file cells-14-01389-s001.zip › cells-3844364-supplementary.pdf]

Supplementary Table S1. Information of *miR-21* polymorphisms for population diversity frequency

Population	N	***miR-21*** **rs1292037 T>C**			**Allele frequencies**		Reference	N	***miR-21*** **rs13137 A>T**			**Allele frequencies**		Reference
		**TT (%)**	**TC (%)**	**CC (%)**	**T**	**C**			**AA (%)**	**AT (%)**	**TT (%)**	**A**	**T**	
East Asian	504	160 (31.8)	227 (45.0)	117 (23.2)	0.543	0.457		504	160 (31.8)	227 (45.0)	117 (23.2)	0.543	0.457	
Europe	503	337 (67.0)	149 (29.6)	17 (3.4)	0.818	0.182		503	337 (67.0)	149 (29.6)	17 (3.4)	0.818	0.182	
African	661	566 (85.6)	90 (13.6)	5 (0.8)	0.924	0.076	1000 Genomes Project	661	566 (85.6)	90 (13.6)	5 (0.8)	0.924	0.076	1000 Genomes Project
American	347	254 (73.2)	81 (23.3)	12 (3.5)	0.849	0.151		347	254 (73.2)	81 (23.3)	12 (3.5)	0.849	0.151	
South Asian	489	179 (36.6)	232 (47.4)	78 (16.0)	0.603	0.397		489	179 (36.6)	232 (47.4)	78 (16.0)	0.603	0.397	
Korean	1465	351 (24.0)	732 (50.0)	382 (26.1)	0.489	0.511	KRGDB	1465	351 (24.0)	732 (50.0)	382 (26.1)	0.489	0.511	KRGDB
Korean	916	224 (24.5)	458 (50.0)	234 (25.6)	0.495	0.506	Korea1K	916	224 (24.5)	458 (50.0)	234 (25.6)	0.495	0.506	Korea1K
Present study	400	102 (25.5)	187 (46.8)	111 (27.8)	0.489	0.511		400	99 (24.8)	208 (52.0)	93 (23.3)	0.508	0.493	
Host gene	VMP1 (vacuole membrane protein 1)													

Note: Data base for NCBI (National Center for Biotechnology Information) and ensembl genome browser.

Supplementary Table S2. Information of *miR-26a* polymorphism for population diversity frequency

Population	N	*miR-26a* rs7372209 C>T			Allele frequencies		Reference
		CC(%)	CT(%)	TT(%)	C	T	
East Asian	504	267 (53.0)	200 (39.6)	37 (7.4)	0.728	0.272	
Europe	503	245 (48.7)	212 (42.2)	46 (9.1)	0.698	0.302	
African	661	636 (96.3)	25 (3.7)	0 (0.0)	0.981	0.019	1000 Genomes Project
American	347	134 (38.6)	163 (47.1)	50 (14.4)	0.621	0.379	
South Asian	489	357 (72.9)	122 (24.9)	10 (2.1)	0.854	0.146	
Korean	1465	830 (56.7)	545 (37.2)	89 (6.1)	0.753	0.247	KRGDB
Present study	400	214 (53.5)	155 (38.8)	31 (7.8)	0.729	0.271	
Host gene	CTDSPL (CTD small phosphatase like)						

Note: Data base for NCBI (National Center for Biotechnology Information) and ensembl genome browser.

Supplementary Table S3. Information of *miR-107* and *124-1* polymorphism for population diversity frequency

Population	N	*miR-107* rs2296616 A>G			Allele frequencies		Reference	N	*miR-124-1* rs531564 G>C			Allele frequencies		Reference
		AA (%)	AG (%)	GG (%)	A	G			GG (%)	GC (%)	CC (%)	G	C	
East Asian	504	448 (88.9)	55 (10.9)	1 (0.2)	0.944	0.057		504	350 (69.4)	146 (29.0)	8 (1.6)	0.839	0.161	
Europe	503	141 (28.0)	278 (55.3)	84 (16.7)	0.557	0.443		503	393 (78.1)	103 (20.5)	7 (1.4)	0.884	0.116	
African	661	323 (48.8)	292 (44.2)	46 (7.0)	0.710	0.291	1000 Genomes Project	661	453 (68.5)	169 (25.6)	39 (5.9)	0.813	0.187	1000 Genomes Project
American	347	143 (41.2)	153 (44.1)	51 (14.7)	0.633	0.367		347	288 (83.0)	56 (16.1)	3 (0.9)	0.911	0.089	
South Asian	489	209 (42.7)	212 (43.4)	68 (13.9)	0.644	0.356		489	426 (87.1)	63 (12.9)	0 (0.0)	0.836	0.064	
Korean	1465	1211 (82.7)	242 (16.5)	12 (0.8)	0.909	0.091	KRGDB	1465	1071 (73.1)	363 (24.8)	31 (2.1)	0.855	0.145	KRGDB
Present study	400	329 (82.3)	66 (16.5)	5 (1.3)	0.905	0.095		400	302 (75.5)	88 (22.0)	10 (2.5)	0.865	0.135	
Host gene	PANK1 (pantothenate kinase 1)							MIR124-1HG (MIR124-1 host gene)						

Note: Data base for NCBI (National Center for Biotechnology Information) and ensembl genome browser.

Supplementary Table S4. Information of miR-*126* polymorphism for population diversity frequency

Population	N	*miR-126* rs4636297 G>A			Allele frequencies		Reference
		GG (%)	GA (%)	AA (%)	G	A	
East Asian	504	332 (65.9)	156 (31.0)	16 (3.2)	0.813	0.187	
Europe	503	207 (41.2)	226 (44.9)	70 (13.9)	0.636	0.364	
African	661	265 (40.1)	309 (46.7)	87 (13.2)	0.635	0.365	1000 Genomes Project
American	347	139 (40.1)	170 (49.0)	38 (11.0)	0.646	0.354	
South Asian	489	280 (57.3)	170 (34.8)	39 (8.0)	0.746	0.254	
Korean	1465	1104 (75.3)	336 (22.9)	26 (1.7)	0.868	0.132	KRGDB
Present study	400	296 (74.0)	93 (23.3)	11 (2.8)	0.856	0.144	
Host gene	EGFL7 (EGF like domain multiple 7)						

Note: Data base for NCBI (National Center for Biotechnology Information) and ensembl genome browser.

Supplementary Table S5. Stratified analysis between the six miRNA genotypes and clinical factors of ischemic stroke

Characteristics	*miR-21* rs1292037 T>C Recessive (TT+TC vs. CC)		*miR-21* rs13137 A>T Recessive (AA+AT vs. TT)		*miR-26a* rs7372209 C>T Recessive (CC+CT vs. TT)	
	AOR (95% CI)*	*P*	AOR (95% CI)*	*P*	AOR (95% CI)*	*P*
Age						
<63	0.791 (0.497 - 1.259)	0.323	0.783 (0.467 - 1.312)	0.353	0.474 (0.207 - 1.084)	0.077
≥63	0.899 (0.588 - 1.375)	0.624	0.716 (0.454 - 1.127)	0.149	**2.361 (1.163 - 4.791)**	**0.017**
Sex						
Male	0.932 (0.571 - 1.521)	0.779	0.867 (0.499 - 1.506)	0.612	0.886 (0.393 - 1.998)	0.771
Female	0.793 (0.526 - 1.198)	0.271	0.655 (0.422 - 1.016)	0.059	1.423 (0.729 - 2.777)	0.301
Hypertension						
No	1.193 (0.754 - 1.887)	0.451	1.051 (0.634 - 1.743)	0.848	0.978 (0.502 - 1.908)	0.949
Yes	**0.619 (0.407 - 0.941)**	**0.025**	**0.539 (0.345 - 0.841)**	**0.007**	1.807 (0.751 - 4.352)	0.187
Diabetes mellitus						
No	0.733 (0.517 - 1.039)	0.081	**0.667 (0.454 - 0.979)**	**0.039**	1.071 (0.611 - 1.875)	0.811
Yes	1.465 (0.696 - 3.087)	0.315	0.954 (0.452 - 2.013)	0.902	2.828 (0.597 - 13.388)	0.190
Hyperlipidemia						
No	0.844 (0.590 - 1.209)	0.356	0.695 (0.469 - 1.028)	0.068	1.097 (0.604 - 1.992)	0.761
Yes	0.811 (0.431 - 1.526)	0.515	0.820 (0.414 - 1.622)	0.568	1.717 (0.623 - 4.730)	0.296
Smoking						
No	0.752 (0.507 - 1.117)	0.158	**0.643 (0.422 - 0.981)**	**0.040**	1.374 (0.738 - 2.561)	0.317
Yes	0.961 (0.571 - 1.619)	0.882	0.838 (0.467 - 1.505)	0.555	0.917 (0.368 - 2.283)	0.852
HDL-c						
≥ 40(M)/50(F)	0.764 (0.439 - 1.329)	0.341	0.752 (0.411 - 1.376)	0.356	6.478 (0.820 - 51.182)	0.076
< 40(M)/50(F)	0.731 (0.410 - 1.304)	0.289	0.933 (0.465 - 1.871)	0.846	1.118 (0.433 - 2.889)	0.818
LDL-c						
< 130 mg/dL	0.752 (0.467 - 1.210)	0.240	0.944 (0.545 - 1.635)	0.838	1.650 (0.648 - 4.204)	0.294
≥ 130 mg/dL	0.707 (0.341 - 1.469)	0.353	0.623 (0.283 - 1.375)	0.242	2.208 (0.459 - 10.624)	0.323
Folate †						
> 3.55 nmol/L	0.979 (0.700 - 1.371)	0.904	0.853 (0.595 - 1.223)	0.386	1.321 (0.768 - 2.273)	0.315
≤ 3.55 nmol/L	**0.317 (0.114 - 0.881)**	**0.028**	**0.155 (0.042 - 0.569)**	**0.005**	0.500 (0.088 - 2.836)	0.434
Homocysteine ‡						
< 13.6 μmol/L	0.787 (0.563 - 1.100)	0.161	0.733 (0.510 - 1.052)	0.092	1.079 (0.622 - 1.870)	0.787
≥ 13.6 μmol/L	1.298 (0.503 - 3.352)	0.590	0.663 (0.226 - 1.946)	0.454	2.399 (0.459 - 12.535)	0.300

AOR, adjusted odds ratio; 95% CI, 95% confidence interval; HDL-c, high density lipoprotein-cholesterol; LDL-c, low density lipoprotein-cholesterol. *P*-values <0.05 are bold

* The adjusted odds ratio on the basis of risk factors, such as age, gender, hypertension, diabetes mellitus, hyperlipidemia, smoking.

† Folate 3.55 nmol/L was lower 15% cut-off each level in ischemic stroke patients and controls.

‡ Homocysteine 13.6 umol/L was upper 15% cut-off each level in ischemic stroke patients and controls.

Supplementary Table S5. *Cont.*

Characteristics	*miR-21* rs1292037 T>C Recessive (TT+TC vs. CC)		*miR-21* rs13137 A>T Recessive (AA+AT vs. TT)		*miR-26a* rs7372209 C>T Recessive (CC+CT vs. TT)	
	AOR(95% CI)*	*P*	AOR(95% CI)*	*P*	AOR(95% CI)*	*P*
Platelet ‡						
< 305 $10^3/\mu\ell$	0.854 (0.608 - 1.200)	0.362	0.796 (0.551 - 1.149)	0.223	1.243 (0.730 - 2.116)	0.424
≥ 305 $10^3/\mu\ell$	0.790 (0.346 - 1.800)	0.574	0.538 (0.207 - 1.399)	0.203	1.034 (0.126 - 8.521)	0.975
PT †						
> 11.00 sec	0.733 (0.504 - 1.065)	0.103	0.720 (0.476 - 1.089)	0.119	1.320 (0.711 - 2.452)	0.379
≤ 11.00 sec	0.560 (0.247 - 1.271)	0.165	0.510 (0.210 - 1.240)	0.137	0.688 (0.136 - 3.481)	0.651
aPTT †						
> 26.40 sec	**0.650 (0.450 - 0.939)**	**0.022**	**0.668 (0.447 - 0.999)**	**0.050**	0.862 (0.466 - 1.594)	0.635
≤ 26.40 sec	1.057 (0.412 - 2.711)	0.909	0.873 (0.300 - 2.543)	0.804	**16.309 (1.806 - 147.259)**	**0.013**
Fibrinogen ‡						
< 542 mg/dl	**0.629 (0.396 - 0.999)**	**0.049**	1.014 (0.585 - 1.759)	0.960	1.505 (0.596 - 3.798)	0.387
≥ 542 mg/dl	**6.504 (1.189 - 35.566)**	**0.031**	1.478 (0.348 - 6.285)	0.597	2.949 (0.295 - 29.473)	0.357
Total cholesterol ‡						
< 228 mg/dl	0.812 (0.579 - 1.140)	0.229	0.747 (0.516 - 1.081)	0.121	1.217 (0.703 - 2.105)	0.483
≥ 228 mg/dl	0.941 (0.374 - 2.369)	0.898	0.523 (0.186 - 1.472)	0.219	0.661 (0.123 - 3.542)	0.629
Triglyceride ‡						
< 219 mg/dl	0.844 (0.602 - 1.183)	0.326	**0.684 (0.473 - 0.989)**	**0.043**	1.099 (0.622 - 1.942)	0.746
≥ 219 mg/dl	0.787 (0.305 - 2.031)	0.620	1.098 (0.396 - 3.048)	0.857	3.653 (0.923 - 14.458)	0.065
Vitamin B12 †						
> 426 pg/mL	0.882 (0.628 - 1.239)	0.469	0.785 (0.542 - 1.138)	0.201	1.246 (0.731 - 2.125)	0.419
≤ 426 pg/mL	0.689 (0.294 - 1.611)	0.390	0.514 (0.208 - 1.274)	0.151	1.215 (0.165 - 8.951)	0.848
FBS						
< 100 mg/dL	0.918 (0.480 - 1.755)	0.796	0.627 (0.303 - 1.297)	0.208	1.112 (0.413 - 2.993)	0.834
≥ 100 mg/dL	0.786 (0.543 - 1.138)	0.202	0.742 (0.496 - 1.109)	0.145	1.366 (0.710 - 2.631)	0.351
BUN ‡						
< 20.7 mg/dl	0.814 (0.580 - 1.143)	0.235	0.697 (0.482 - 1.010)	0.057	1.108 (0.630 - 1.950)	0.721
≥ 20.7 mg/dl	1.144 (0.488 - 2.681)	0.757	1.088 (0.433 - 2.734)	0.858	2.647 (0.650 - 10.774)	0.174
Uric acid ‡						
< 6.1 mg/dl	0.870 (0.620 - 1.221)	0.421	0.735 (0.512 - 1.057)	0.097	1.255 (0.701 - 2.245)	0.444
≥ 6.1 mg/dl	0.685 (0.270 - 1.738)	0.426	0.803 (0.243 - 2.659)	0.720	1.456 (0.440 - 4.817)	0.538

AOR, adjusted odds ratio; 95% CI, 95% confidence interval; FBS, fasting blood sugar; PT, prothrombin time; aPTT, activated partial thromboplastin time. *P*-values <0.05 are bold

* The adjusted odds ratio on the basis of risk factors, such as age, gender, hypertension, diabetes mellitus, hyperlipidemia, smoking.

† Vitamin B12 426 pg/mL, PT 11.00 sec, and aPTT 26.30 sec were lower 15% cut-off each level in ischemic stroke patients and controls.

‡ Platelet 305 $10^3/\mu\ell$, fibrinogen 542 mg/dl, total cholesterol 228 mg/dl, triglyceride 219 mg/dl, BUN 20.7 mg/dl, and uric acid 6.1 mg/dl were upper 15% cut-off each level in ischemic stroke patients and controls.

Supplementary Table S5. *Cont.*

Characteristics	*miR-107* rs2296616 A>G Dominant (AA vs. AG+GG)		*miR-124-1* rs531564 G>C Dominant (GG vs. GC+CC)		*miR-126* rs4636297 G>A Dominant (GG vs. GA+AA)	
	AOR (95% CI)*	*P*	AOR (95% CI)*	*P*	AOR (95% CI)*	*P*
Age						
<63	0.794 (0.464 - 1.359)	0.400	1.233 (0.769 - 1.978)	0.384	**1.581 (1.001 - 2.497)**	**0.050**
≥63	1.399 (0.856 - 2.287)	0.181	1.141 (0.746 - 1.744)	0.543	1.495 (0.984 - 2.270)	0.059
Sex						
Male	0.937 (0.536 - 1.637)	0.820	1.041 (0.639 - 1.699)	0.871	**1.853 (1.153 - 2.980)**	**0.011**
Female	1.115 (0.693 - 1.794)	0.655	1.331 (0.880 - 2.014)	0.176	1.337 (0.888 - 2.014)	0.164
Hypertension						
No	0.911 (0.547 - 1.516)	0.719	1.002 (0.634 - 1.581)	0.995	1.232 (0.804 - 1.889)	0.338
Yes	1.270 (0.753 - 2.145)	0.371	1.421 (0.911 - 2.215)	0.121	**1.996 (1.261 - 3.161)**	**0.003**
Diabetes mellitus						
No	1.190 (0.803 - 1.762)	0.386	1.354 (0.952 - 1.925)	0.092	**1.447 (1.028 - 2.037)**	**0.034**
Yes	0.655 (0.279 - 1.539)	0.332	0.736 (0.369 - 1.469)	0.385	1.986 (0.961 - 4.102)	0.064
Hyperlipidemia						
No	1.150 (0.761 - 1.738)	0.508	1.323 (0.926 - 1.891)	0.125	**1.851 (1.299 - 2.637)**	**0.001**
Yes	0.861 (0.422 - 1.755)	0.680	0.846 (0.442 - 1.619)	0.613	0.839 (0.455 - 1.547)	0.574
Smoking						
No	1.178 (0.746 - 1.862)	0.483	1.029 (0.695 - 1.523)	0.886	1.426 (0.974 - 2.087)	0.068
Yes	0.826 (0.459 - 1.487)	0.525	1.546 (0.900 - 2.655)	0.115	**1.768 (1.043 - 2.996)**	**0.034**
HDL-c						
≥ 40(M)/50(F)	1.162 (0.588 - 2.298)	0.666	1.026 (0.566 - 1.860)	0.933	1.449 (0.815 - 2.577)	0.207
< 40(M)/50(F)	0.872 (0.454 - 1.676)	0.681	1.211 (0.679 - 2.160)	0.516	**2.783 (1.443 - 5.370)**	**0.002**
LDL-c						
< 130 mg/dL	1.113 (0.609 - 2.033)	0.728	1.161 (0.714 - 1.887)	0.548	**1.921 (1.151 - 3.207)**	**0.013**
≥ 130 mg/dL	0.760 (0.341 - 1.693)	0.502	1.134 (0.517 - 2.489)	0.754	1.877 (0.871 - 4.045)	0.108
Folate †						
> 3.55 nmol/L	1.059 (0.716 - 1.567)	0.775	1.096 (0.780 - 1.540)	0.599	**1.620 (1.158 - 2.267)**	**0.005**
≤ 3.55 nmol/L	0.918 (0.315 - 2.672)	0.875	1.950 (0.671 - 5.664)	0.220	0.866 (0.351 - 2.132)	0.754
Homocysteine ‡						
< 13.6 μmol/L	0.956 (0.649 - 1.409)	0.821	1.256 (0.890 - 1.773)	0.194	1.372 (0.986 - 1.909)	0.061
≥ 13.6 μmol/L	2.233 (0.741 - 6.726)	0.153	0.807 (0.352 - 1.847)	0.611	**2.846 (1.151 - 7.035)**	**0.024**

AOR, adjusted odds ratio; 95% CI, 95% confidence interval; HDL-c, high density lipoprotein-cholesterol; LDL-c, low density lipoprotein-cholesterol. *P*-values <0.05 are bold

* The adjusted odds ratio on the basis of risk factors, such as age, gender, hypertension, diabetes mellitus, hyperlipidemia, smoking.

† Folate 3.55 nmol/L was lower 15% cut-off each level in ischemic stroke patients and controls.

‡ Homocysteine 13.6 umol/L was upper 15% cut-off each level in ischemic stroke patients and controls.

Supplementary Table S5. *Cont.*

Characteristics	*miR-107* rs2296616 A>G Dominant (AA vs. AG+GG)		*miR-124-1* rs531564 G>C Dominant (GG vs. GC+CC)		*miR-126* rs4636297 G>A Dominant (GG vs. GA+AA)	
	AOR (95% CI)*	*P*	AOR (95% CI)*	*P*	AOR (95% CI)*	*P*
Platelet ‡						
< 305 $10^3/\mu\ell$	1.144 (0.768 - 1.703)	0.508	1.090 (0.773 - 1.536)	0.624	**1.581 (1.135 - 2.201)**	**0.007**
≥ 305 $10^3/\mu\ell$	0.804 (0.338 - 1.910)	0.621	2.011 (0.880 - 4.594)	0.098	1.181 (0.502 - 2.780)	0.703
PT †						
> 11.00 sec	1.029 (0.675 - 1.568)	0.896	1.110 (0.758 - 1.625)	0.592	**1.622 (1.114 - 2.361)**	**0.012**
≤ 11.00 sec	0.703 (0.252 - 1.967)	0.502	1.138 (0.493 - 2.624)	0.762	2.147 (0.863 - 5.340)	0.100
aPTT †						
> 26.40 sec	1.012 (0.654 - 1.568)	0.956	1.341 (0.913 - 1.970)	0.134	**1.493 (1.027 - 2.170)**	**0.036**
≤ 26.40 sec	0.578 (0.217 - 1.544)	0.274	0.485 (0.199 - 1.182)	0.112	2.252 (0.885 - 5.730)	0.088
Fibrinogen ‡						
< 542 mg/dl	1.020 (0.589 - 1.766)	0.944	1.397 (0.848 - 2.303)	0.190	1.559 (0.972 - 2.500)	0.066
≥ 542 mg/dl	1.128 (0.314 - 4.046)	0.854	0.898 (0.306 - 2.638)	0.845	2.815 (0.731 - 10.838)	0.133
Total cholesterol ‡						
< 228 mg/dl	1.017 (0.687 - 1.506)	0.932	1.236 (0.877 - 1.742)	0.226	**1.468 (1.054 - 2.046)**	**0.023**
≥ 228 mg/dl	1.294 (0.483 - 3.467)	0.608	1.013 (0.436 - 2.356)	0.976	1.683 (0.668 - 4.238)	0.270
Triglyceride ‡						
< 219 mg/dl	1.031 (0.698 - 1.523)	0.879	1.298 (0.925 - 1.820)	0.132	**1.660 (1.186 - 2.324)**	**0.003**
≥ 219 mg/dl	1.018 (0.360 - 2.879)	0.973	0.587 (0.228 - 1.514)	0.271	0.909 (0.379 - 2.181)	0.830
Vitamin B12 †						
> 426 pg/mL	1.087 (0.737 - 1.602)	0.674	1.184 (0.847 - 1.654)	0.324	1.384 (0.994 - 1.928)	0.055
≤ 426 pg/mL	0.892 (0.337 - 2.359)	0.817	1.395 (0.517 - 3.766)	0.511	**2.505 (1.053 - 5.959)**	**0.038**
FBS						
< 100 mg/dL	1.320 (0.674 - 2.585)	0.418	1.136 (0.623 - 2.070)	0.678	1.336 (0.737 - 2.423)	0.340
≥ 100 mg/dL	1.027 (0.663 - 1.591)	0.906	1.197 (0.816 - 1.756)	0.359	**1.558 (1.074 - 2.261)**	**0.020**
BUN ‡						
< 20.7 mg/dl	1.126 (0.760 - 1.668)	0.555	1.277 (0.906 - 1.800)	0.163	**1.584 (1.136 - 2.208)**	**0.007**
≥ 20.7 mg/dl	0.793 (0.310 - 2.030)	0.629	0.644 (0.275 - 1.513)	0.313	1.193 (0.502 - 2.836)	0.689
Uric acid ‡						
< 6.1 mg/dl	1.054 (0.715 - 1.554)	0.790	1.255 (0.888 - 1.773)	0.199	1.368 (0.980 - 1.910)	0.066
≥ 6.1 mg/dl	0.657 (0.227 - 1.899)	0.438	0.882 (0.361 - 2.153)	0.782	2.335 (0.974 - 5.599)	0.057

AOR, adjusted odds ratio; 95% CI, 95% confidence interval; FBS, fasting blood sugar; PT, prothrombin time; aPTT, activated partial thromboplastin time. *P*-values <0.05 are bold

* The adjusted odds ratio on the basis of risk factors, such as age, gender, hypertension, diabetes mellitus, hyperlipidemia, smoking.

† Vitamin B12 426 pg/mL, PT 11.00 sec, and aPTT 26.30 sec were lower 15% cut-off each level in ischemic stroke patients and controls.

‡ Platelet 305 $10^3/\mu\ell$, fibrinogen 542 mg/dl, total cholesterol 228 mg/dl, triglyceride 219 mg/dl, BUN 20.7 mg/dl, and uric acid 6.1 mg/dl were upper 15% cut-off each level in ischemic stroke patients and controls.

Supplementary Table S6. Ischemic stroke prevalence analyses between the six miRNA polymorphisms and several environmental factors using interaction analysis

Characteristics	*miR-21* rs1292037 T>C TT	*miR-21* rs1292037 T>C TC+CC	*miR-21* rs13137 A>T AA	*miR-21* rs13137 A>T AT+TT	*miR-26a* rs7372209 C>T CC	*miR-26a* rs7372209 C>T CT+TT
	AOR (95% CI)*	AOR (95% CI)*	AOR (95% CI)*	AOR (95% CI)*	AOR (95% CI)*	AOR (95% CI)*
Age						
<63	1.000 (reference)	1.059 (0.647 - 1.736)	1.000 (reference)	0.723 (0.445 - 1.174)	1.000 (reference)	1.008 (0.667 - 1.524)
≥63	0.593 (0.302 - 1.163)	0.786 (0.475 - 1.302)	0.671 (0.357 - 1.260)	**0.550 (0.333 - 0.907)**	0.791 (0.525 - 1.192)	0.931 (0.620 - 1.399)
Sex						
Male	1.000 (reference)	1.426 (0.867 - 2.345)	1.000 (reference)	0.863 (0.534 - 1.393)	1.000 (reference)	0.961 (0.630 - 1.465)
Female	1.410 (0.633 - 3.141)	0.971 (0.553 - 1.704)	1.010 (0.485 - 2.104)	**0.571 (0.331 - 0.983)**	0.822 (0.509 - 1.326)	1.052 (0.650 - 1.703)
Hypertension						
No	1.000 (reference)	**2.117 (1.300 - 3.448)**	1.000 (reference)	1.362 (0.866 - 2.143)	1.000 (reference)	1.278 (0.856 - 1.908)
Yes	**6.227 (3.389 - 11.439)**	**4.005 (2.491 - 6.438)**	**5.715 (3.198 - 10.211)**	**2.631 (1.699 - 4.075)**	**3.104 (2.084 - 4.625)**	**2.785 (1.847 - 4.199)**
Diabetes mellitus						
No	1.000 (reference)	1.206 (0.846 - 1.720)	1.000 (reference)	0.864 (0.613 - 1.216)	1.000 (reference)	1.036 (0.763 - 1.406)
Yes	**2.581 (1.166 - 5.712)**	**2.265 (1.396 - 3.676)**	**3.183 (1.388 - 7.298)**	1.524 (0.957 - 2.428)	**1.703 (1.065 - 2.722)**	**2.406 (1.345 - 4.305)**
Hyperlipidemia						
No	1.000 (reference)	1.045 (0.725 - 1.508)	1.000 (reference)	0.740 (0.515 - 1.062)	1.000 (reference)	1.245 (0.907 - 1.711)
Yes	1.097 (0.545 - 2.208)	**1.715 (1.077 - 2.731)**	1.232 (0.641 - 2.367)	1.162 (0.724 - 1.864)	**2.002 (1.272 - 3.152)**	1.463 (0.927 - 2.310)
Smoking						
No	1.000 (reference)	1.047 (0.702 - 1.563)	1.000 (reference)	0.696 (0.469 - 1.032)	1.000 (reference)	1.210 (0.855 - 1.715)
Yes	1.692 (0.790 - 3.625)	1.377 (0.819 - 2.316)	1.345 (0.672 - 2.693)	0.833 (0.493 - 1.406)	1.524 (0.945 - 2.459)	1.564 (0.973 - 2.514)
HDL-c						
≥ 40(M)/50(F)	1.000 (reference)	0.897 (0.477 - 1.687)	1.000 (reference)	0.644 (0.343 - 1.208)	1.000 (reference)	1.180 (0.705 - 1.974)
< 40(M)/50(F)	1.221 (0.540 - 2.762)	1.649 (0.859 - 3.167)	1.647 (0.709 - 3.829)	1.082 (0.568 - 2.059)	**1.656 (1.009 - 2.716)**	**2.112 (1.245 - 3.581)**
LDL-c						
< 130 mg/dL	1.000 (reference)	1.133 (0.690 - 1.859)	1.000 (reference)	0.656 (0.392 - 1.097)	1.000 (reference)	1.214 (0.782 - 1.886)
≥ 130 mg/dL	1.868 (0.729 - 4.785)	1.448 (0.793 - 2.643)	1.569 (0.617 - 3.990)	0.947 (0.514 - 1.744)	1.242 (0.715 - 2.158)	**1.943 (1.040 - 3.629)**
Folate †						
> 3.55 nmol/L	1.000 (reference)	1.248 (0.882 - 1.765)	1.000 (reference)	0.899 (0.642 - 1.258)	1.000 (reference)	1.105 (0.819 - 1.490)
≤ 3.55 nmol/L	**6.846 (2.407 - 19.467)**	**4.656 (2.471 - 8.774)**	**9.423 (3.244 - 27.370)**	**2.753 (1.496 - 5.068)**	**4.827 (2.263 - 10.295)**	**2.892 (1.594 - 5.245)**
Homocysteine ‡						
< 13.6 μmol/L	1.000 (reference)	1.174 (0.832 - 1.658)	1.000 (reference)	0.835 (0.597 - 1.168)	1.000 (reference)	1.137 (0.844 - 1.531)
≥ 13.6 μmol/L	2.417 (0.984 - 5.939)	**1.829 (1.038 - 3.222)**	**3.087 (1.264 - 7.538)**	1.165 (0.660 - 2.056)	**1.807 (1.015 - 3.216)**	1.511 (0.847 - 2.695)

AOR, adjusted odds ratio; 95% CI, 95% confidence interval; HDL-c, high density lipoprotein-cholesterol; LDL-c, low density lipoprotein-cholesterol. *P*-values <0.05 are bold

* The adjusted odds ratio on the basis of risk factors, such as age, gender, hypertension, diabetes mellitus, hyperlipidemia, smoking.

† Folate 3.55 nmol/L was lower 15% cut-off each level in ischemic stroke patients and controls.

‡ Homocysteine 13.6 umol/L was upper 15% cut-off each level in ischemic stroke patients and controls.

Supplementary Table S6. *Cont.*

Characteristics	*miR-21* rs1292037 T>C TT	*miR-21* rs1292037 T>C TC+CC	*miR-21* rs13137 A>T AA	*miR-21* rs13137 A>T AT+TT	*miR-26a* rs7372209 C>T CC	*miR-26a* rs7372209 C>T CT+TT
	AOR (95% CI)*	AOR (95% CI)*	AOR (95% CI)*	AOR (95% CI)*	AOR (95% CI)*	AOR (95% CI)*
Platelet ‡						
< 305 $10^3/\mu\ell$	1.000 (reference)	1.202 (0.852 - 1.696)	1.000 (reference)	0.795 (0.568 - 1.113)	1.000 (reference)	1.191 (0.883 - 1.608)
≥305 $10^3/\mu\ell$	1.052 (0.429 - 2.579)	0.980 (0.578 - 1.661)	0.808 (0.351 - 1.860)	0.676 (0.397 - 1.153)	1.153 (0.671 - 1.982)	0.828 (0.463 - 1.480)
PT †						
> 11.00 sec	1.000 (reference)	0.939 (0.633 - 1.394)	1.000 (reference)	**0.633 (0.427 - 0.938)**	1.000 (reference)	1.043 (0.746 - 1.458)
≤ 11.00 sec	0.718 (0.301 - 1.710)	0.943 (0.529 - 1.682)	0.799 (0.348 - 1.831)	0.597 (0.334 - 1.067)	0.965 (0.552 - 1.687)	0.987 (0.540 - 1.801)
aPTT †						
> 26.40 sec	1.000 (reference)	0.993 (0.669 - 1.474)	1.000 (reference)	0.697 (0.471 - 1.029)	1.000 (reference)	0.950 (0.681 - 1.325)
≤ 26.40 sec	0.950 (0.379 - 2.381)	0.760 (0.421 - 1.369)	1.061 (0.444 - 0.253)	**0.492 (0.268 - 0.903)**	0.654 (0.369 - 1.162)	1.190 (0.627 - 2.256)
Fibrinogen ‡						
< 542 mg/dl	1.000 (reference)	0.922 (0.564 - 1.508)	1.000 (reference)	0.639 (0.390 - 1.045)	1.000 (reference)	1.368 (0.893 - 2.097)
≥ 542 mg/dl	0.324 (0.086 - 1.216)	0.946 (0.454 - 1.969)	0.505 (0.135 - 1.889)	0.661 (0.317 - 1.378)	0.883 (0.422 - 1.850)	1.276 (0.564 - 2.888)
Total cholesterol ‡						
< 228 mg/dl	1.000 (reference)	1.109 (0.784 - 1.569)	1.000 (reference)	0.781 (0.554 - 1.099)	1.000 (reference)	1.178 (0.872 - 1.592)
≥ 228 mg/dl	0.546 (0.205 - 1.453)	0.682 (0.343 - 1.359)	0.516 (0.210 - 1.265)	**0.442 (0.221 - 0.884)**	0.752 (0.402 - 1.407)	0.542 (0.274 - 1.072)
Triglyceride ‡						
< 219 mg/dl	1.000 (reference)	1.143 (0.810 - 1.614)	1.000 (reference)	0.838 (0.598 - 1.173)	1.000 (reference)	1.114 (0.826 - 1.503)
≥ 219 mg/dl	0.519 (0.187 - 1.443)	0.614 (0.289 - 1.304)	0.656 (0.245 - 1.758)	**0.374 (0.181 - 0.771)**	**0.468 (0.234 - 0.934)**	**0.465 (0.228 - 0.949)**
Vitamin B12 †						
> 426 pg/mL	1.000 (reference)	1.277 (0.903 - 1.807)	1.000 (reference)	0.871 (0.622 - 1.219)	1.000 (reference)	1.103 (0.818 - 1.487)
≤ 426 pg/mL	2.071 (0.893 - 4.802)	1.448 (0.849 - 2.470)	1.876 (0.837 - 4.202)	0.946 (0.556 - 1.608)	1.292 (0.776 - 2.152)	1.493 (0.803 - 2.778)
FBS						
< 100 mg/dL	1.000 (reference)	**2.185 (1.161 - 4.113)**	1.000 (reference)	1.268 (0.710 - 2.267)	1.000 (reference)	1.024 (0.597 - 1.756)
≥ 100 mg/dL	**4.405 (2.222 - 8.734)**	**3.890 (2.228 - 6.793)**	**3.889 (2.090 - 7.237)**	**2.412 (1.464 - 3.973)**	**2.120 (1.359 - 3.308)**	**2.607 (1.665 - 4.080)**
BUN ‡						
< 20.7 mg/dl	1.000 (reference)	1.171 (0.832 - 1.648)	1.000 (reference)	0.817 (0.585 - 1.142)	1.000 (reference)	1.037 (0.769 - 1.400)
≥ 20.7 mg/dl	1.816 (0.685 - 4.818)	1.476 (0.876 - 2.486)	1.872 (0.747 - 4.694)	0.967 (0.572 - 1.635)	1.039 (0.599 - 1.805)	**1.959 (1.075 - 3.569)**
Uric acid ‡						
< 6.1 mg/dl	1.000 (reference)	1.150 (0.815 - 1.622)	1.000 (reference)	0.831 (0.594 - 1.164)	1.000 (reference)	1.224 (0.904 - 1.658)
≥ 6.1 mg/dl	0.653 (0.256 - 1.6964)	0.882 (0.500 - 1.555)	0.827 (0.338 - 2.028)	0.614 (0.347 - 1.084)	1.015 (0.572 - 1.800)	0.667 (0.369 - 1.206)

AOR, adjusted odds ratio; 95% CI, 95% confidence interval; FBS, fasting blood sugar; PT, prothrombin time; aPTT, activated partial thromboplastin time. *P*-values <0.05 are bold

* The adjusted odds ratio on the basis of risk factors, such as age, gender, hypertension, diabetes mellitus, hyperlipidemia, smoking.

† Vitamin B12 426 pg/mL, PT 11.00 sec, and aPTT 26.30 sec were lower 15% cut-off each level in ischemic stroke patients and controls.

‡ Platelet 305 $10^3/\mu\ell$, fibrinogen 542 mg/dl, total cholesterol 228 mg/dl, triglyceride 219 mg/dl, BUN 20.7 mg/dl, and uric acid 6.1 mg/dl were upper 15% cut-off each level in ischemic stroke patients and controls.

Supplementary Table S6. *Cont.*

Characteristics	*miR-107* rs2296616 A>G AA	*miR-107* rs2296616 A>G AG+GG	*miR-124-1* rs531564 G>C GG	*miR-124-1* rs531564 G>C GC+CC	*miR-126* rs4636297 G>A GG	*miR-126* rs4636297 G>A GA+AA
	AOR (95% CI)*	AOR (95% CI)*	AOR (95% CI)*	AOR (95% CI)*	AOR (95% CI)*	AOR (95% CI)*
Age						
<63	1.000 (reference)	0.791 (0.463 - 1.349)	1.000 (reference)	1.264 (0.790 - 2.022)	1.000 (reference)	1.549 (0.980 - 2.447)
≥63	0.780 (0.564 - 1.078)	1.255 (0.746 - 2.109)	0.899 (0.642 - 1.260)	1.096 (0.693 - 1.733)	0.876 (0.620 - 1.239)	1.448 (0.926 - 2.262)
Sex						
Male	1.000 (reference)	0.933 (0.535 - 1.626)	1.000 (reference)	1.058 (0.650 - 1.721)	1.000 (reference)	**1.820 (1.131 - 2.927)**
Female	0.933 (0.649 - 1.343)	1.238 (0.694 - 2.209)	0.767 (0.515 - 1.142)	1.210 (0.741 - 1.976)	1.028 (0.689 - 1.533)	1.615 (0.957 - 2.725)
Hypertension						
No	1.000 (reference)	0.906 (0.546 - 1.505)	1.000 (reference)	1.039 (0.661 - 1.632)	1.000 (reference)	1.212 (0.790 - 1.858)
Yes	**2.430 (1.780 - 3.317)**	**3.035 (1.778 - 5.180)**	**2.327 (1.680 - 3.223)**	**3.385 (2.122 - 5.402)**	**2.329 (1.667 - 3.253)**	**4.456 (2.746 - 7.233)**
Diabetes mellitus						
No	1.000 (reference)	1.184 (0.801 - 1.752)	1.000 (reference)	1.375 (0.968 - 1.952)	1.000 (reference)	**1.422 (1.010 - 2.000)**
Yes	**2.196 (1.483 - 3.251)**	1.479 (0.654 - 3.344)	**2.355 (1.540 - 3.603)**	1.623 (0.877 - 3.006)	**1.700 (1.113 - 2.597)**	**3.705 (1.928 - 7.118)**
Hyperlipidemia						
No	1.000 (reference)	1.146 (0.759 - 1.730)	1.000 (reference)	1.347 (0.944 - 1.922)	1.000 (reference)	**1.822 (1.279 - 2.595)**
Yes	**1.563 (1.096 - 2.228)**	1.357 (0.705 - 2.613)	**1.699 (1.174 - 2.458)**	1.388 (0.766 - 2.515)	**1.868 (1.281 - 2.726)**	1.578 (0.921 - 2.706)
Smoking						
No	1.000 (reference)	1.178 (0.746 - 1.862)	1.000 (reference)	1.029 (0.695 - 1.523)	1.000 (reference)	1.426 (0.974 - 2.087)
Yes	**1.537 (1.068 - 2.213)**	1.576 (0.862 - 2.884)	1.215 (0.822 - 1.798)	**2.035 (1.167 - 3.548)**	1.409 (0.946 - 2.097)	**2.885 (1.634 - 5.095)**
HDL-c						
≥ 40(M)/50(F)	1.000 (reference)	1.149 (0.582 - 2.268)	1.000 (reference)	1.061 (0.587 - 1.918)	1.000 (reference)	1.435 (0.807 - 2.552)
< 40(M)/50(F)	**1.727 (1.149 - 2.596)**	1.532 (0.787 - 2.980)	**1.610 (1.049 - 2.470)**	1.781 (0.989 - 3.209)	1.381 (0.905 - 2.108)	**3.823 (1.964 - 7.441)**
LDL-c						
< 130 mg/dL	1.000 (reference)	1.113 (0.609 - 2.033)	1.000 (reference)	1.161 (0.714 - 1.887)	1.000 (reference)	**1.921 (1.151 - 3.207)**
≥ 130 mg/dL	1.341 (0.850 - 2.117)	1.123 (0.524 - 2.406)	1.291 (0.803 - 2.076)	1.798 (0.856 - 3.779)	1.196 (0.741 - 1.932)	**2.345 (1.156 - 4.761)**
Folate †						
> 3.55 nmol/L	1.000 (reference)	1.057 (0.716 - 1.561)	1.000 (reference)	1.124 (0.802 - 1.577)	1.000 (reference)	**1.583 (1.132 - 2.212)**
≤ 3.55 nmol/L	**3.396 (2.028 - 5.686)**	**3.367 (1.295 - 8.757)**	**3.143 (1.850 - 5.341)**	**5.580 (2.230 - 13.961)**	**4.396 (2.435 - 7.937)**	**3.375 (1.668 - 6.829)**
Homocysteine ‡						
< 13.6 μmol/L	1.000 (reference)	0.960 (0.652 - 1.412)	1.000 (reference)	1.274 (0.904 - 1.795)	1.000 (reference)	1.354 (0.973 - 1.884)
≥ 13.6 μmol/L	1.432 (0.919 - 2.231)	**3.089 (1.123 - 8.493)**	**1.984 (1.202 - 3.276)**	1.446 (0.753 - 2.774	1.430 (0.886 - 2.308)	**3.271 (1.553 - 6.887)**

AOR, adjusted odds ratio; 95% CI, 95% confidence interval; HDL-c, high density lipoprotein-cholesterol; LDL-c, low density lipoprotein-cholesterol. *P*-values <0.05 are bold

* The adjusted odds ratio on the basis of risk factors, such as age, gender, hypertension, diabetes mellitus, hyperlipidemia, smoking.

† Folate 3.55 nmol/L was lower 15% cut-off each level in ischemic stroke patients and controls.

‡ Homocysteine 13.6 umol/L was upper 15% cut-off each level in ischemic stroke patients and controls.

Supplementary Table S6. *Cont.*

Characteristics	*miR-107* rs2296616 A>G AA	*miR-107* rs2296616 A>G AG+GG	*miR-124-1* rs531564 G>C GG	*miR-124-1* rs531564 G>C GC+CC	*miR-126* rs4636297 G>A GG	*miR-126* rs4636297 G>A GA+AA
	AOR (95% CI)*	AOR (95% CI)*	AOR (95% CI)*	AOR (95% CI)*	AOR (95% CI)*	AOR (95% CI)*
Platelet ‡						
< 305 $10^3/\mu\ell$	1.000 (reference)	1.124 (0.756 - 1.672)	1.000 (reference)	1.118 (0.794 - 1.572)	1.000 (reference)	**1.557 (1.118 - 2.167)**
≥305 $10^3/\mu\ell$	0.966 (0.619 - 1.506)	0.839 (0.393 - 1.793)	0.793 (0.501 - 1.256)	1.441 (0.706 - 2.943)	0.994 (0.634 - 1.558)	1.194 (0.568 - 2.509)
PT †						
> 11.00 sec	1.000 (reference)	1.024 (0.672 - 1.560)	1.000 (reference)	1.122 (0.767 - 1.642)	1.000 (reference)	**1.614 (1.109 - 2.350)**
≤ 11.00 sec	1.023 (0.657 - 1.593)	0.642 (0.254 - 1.625)	0.944 (0.581 - 1.532)	1.107 (0.554 - 2.211)	0.930 (0.586 - 1.477)	1.802 (0.824 - 3.941)
aPTT †						
> 26.40 sec	1.000 (reference)	1.003 (0.648 - 1.553)	1.000 (reference)	1.366 (0.931 - 2.005)	1.000 (reference)	**1.481 (1.019 - 2.152)**
≤ 26.40 sec	0.909 (0.563 - 1.468)	0.756 (0.346 - 1.651)	1.131 (0.685 - 1.868)	0.599 (0.287 - 1.252)	0.777 (0.477 - 1.266)	1.707 (0.784 - 3.719)
Fibrinogen ‡						
< 542 mg/dl	1.000 (reference)	1.011 (0.584 - 1.749)	1.000 (reference)	1.426 (0.865 - 2.350)	1.000 (reference)	1.545 (0.964 - 2.477)
≥ 542 mg/dl	0.886 (0.483 - 1.624)	1.127 (0.355 - 3.582)	1.117 (0.569 - 2.192)	0.934 (0.389 - 2.245)	0.841 (0.455 - 1.554)	2.559 (0.738 - 8.873)
Total cholesterol ‡						
< 228 mg/dl	1.000 (reference)	1.013 (0.685 - 1.498)	1.000 (reference)	1.259 (0.895 - 1.772)	1.000 (reference)	**1.446 (1.038 - 2.014)**
≥ 228 mg/dl	**0.597 (0.359 - 0.994)**	0.748 (0.309 - 1.813)	0.649 (0.380 - 1.109)	0.649 (0.310 - 1.359)	**0.516 (0.301 - 0.886)**	1.053 (0.457 - 2.430)
Triglyceride ‡						
< 219 mg/dl	1.000 (reference)	1.029 (0.697 - 1.518)	1.000 (reference)	1.319 (0.942 - 1.848)	1.000 (reference)	**1.635 (1.169 - 2.289)**
≥ 219 mg/dl	**0.538 (0.312 - 0.928)**	0.682 (0.274 - 1.694)	0.666 (0.379 - 1.171)	**0.409 (0.176 - 0.951)**	0.638 (0.359 - 1.134)	0.665 (0.314 - 1.407)
Vitamin B12 †						
> 426 pg/mL	1.000 (reference)	1.077 (0.731 - 1.588)	1.000 (reference)	1.206 (0.864 - 1.684)	1.000 (reference)	1.375 (0.987 - 1.915)
≤ 426 pg/mL	1.352 (0.875 - 2.090)	1.228 (0.537 - 2.808)	1.305 (0.844 - 2.017)	1.743 (0.739 - 4.113)	1.152 (0.727 - 1.827)	**2.492 (1.227 - 5.062)**
FBS						
< 100 mg/dL	1.000 (reference)	1.309 (0.668 - 2.562)	1.000 (reference)	1.161 (0.639 - 2.112)	1.000 (reference)	1.327 (0.731 - 2.407)
≥ 100 mg/dL	**2.364 (1.674 - 3.338)**	**2.371 (1.438 - 3.911)**	**2.262 (1.568 - 3.263)**	**2.692 (1.680 - 4.314)**	**2.226 (1.541 - 3.218)**	**3.197 (2.022 - 5.054)**
BUN ‡						
< 20.7 mg/dl	1.000 (reference)	1.124 (0.760 - 1.663)	1.000 (reference)	1.307 (0.928 - 1.839)	1.000 (reference)	**1.564 (1.122 - 2.179)**
≥ 20.7 mg/dl	1.429 (0.915 - 2.233)	1.231 (0.535 - 2.832)	1.535 (0.952 - 2.476)	1.217 (0.609 - 2.433)	1.483 (0.933 - 2.358)	1.853 (0.876 - 3.922)
Uric acid ‡						
< 6.1 mg/dl	1.000 (reference)	1.057 (0.718 - 1.557)	1.000 (reference)	1.287 (0.912 - 1.815)	1.000 (reference)	1.354 (0.970 - 1.890)
≥ 6.1 mg/dl	0.892 (0.569 - 1.397)	0.658 (0.258 - 1.679)	0.959 (0.582 - 1.581)	0.793 (0.400 - 1.572)	0.757 (0.468 - 1.224)	1.562 (0.741 - 3.296)

AOR, adjusted odds ratio; 95% CI, 95% confidence interval; FBS, fasting blood sugar; PT, prothrombin time; aPTT, activated partial thromboplastin time. *P*-values <0.05 are bold

* The adjusted odds ratio on the basis of risk factors, such as age, gender, hypertension, diabetes mellitus, hyperlipidemia, smoking.

† Vitamin B12 426 pg/mL, PT 11.00 sec, and aPTT 26.30 sec were lower 15% cut-off each level in ischemic stroke patients and controls.

‡ Platelet 305 $10^3/\mu\ell$, fibrinogen 542 mg/dl, total cholesterol 228 mg/dl, triglyceride 219 mg/dl, BUN 20.7 mg/dl, and uric acid 6.1 mg/dl were upper 15% cut-off each level in ischemic stroke patients and controls.

Supplementary Table S7. Comparison of the six miRNA polymorphism frequencies for ischemic stroke LAD patient's survival.

Genotypes	Stroke patients (n=192)	Death patients (n=43)	Adjusted HR (95% CI)*	P†
miR-21 rs1292037 T>C				
TT	44 (22.9)	12 (27.9)	1.000 (reference)	
TC	101 (52.6)	20 (46.5)	0.615 (0.290 - 1.305)	0.208
CC	47 (24.5)	11 (25.6)	0.896 (0.376 - 2.132)	0.804
Dominant (TT vs. TC+CC)			0.709 (0.358 - 1.403)	0.326
Recessive (TT+TC vs. CC)			1.123 (0.561 - 2.248)	0.745
miR-21 rs13137 A>T				
AA	55 (28.6)	13 (30.2)	1.000 (reference)	
AT	98 (51.0)	20 (46.5)	0.776 (0.381 - 1.584)	0.489
TT	39 (20.3)	10 (23.3)	1.072 (0.447 - 2.570)	0.877
Dominant (AA vs. AT+TT)			0.868 (0.447 - 1.684)	0.676
Recessive (AA+AT vs. TT)			1.137 (0.557 - 2.323)	0.726
miR-26a rs7372209C>T				
CC	103 (53.6)	22 (51.2)	1.000 (reference)	
CT	72 (37.5)	16 (37.2)	1.099 (0.573 - 2.108)	0.778
TT	17 (8.9)	5 (11.6)	0.946 (0.333 - 2.686)	0.917
Dominant (CC vs. CT+TT)			1.104 (0.599 - 2.032)	0.753
Recessive (CC+CT vs. TT)			0.903 (0.340 - 2.399)	0.839
miR-107 rs2296616A>G				
AA	159 (82.8)	32 (74.4)	1.000 (reference)	
AG	32 (16.7)	11 (25.6)	**2.296 (1.144 - 4.608)**	**0.020**
GG	1 (0.5)	0 (0.0)	-	0.963
Dominant (AA vs. AG+GG)			**2.057 (1.029 - 4.112)**	**0.042**
Recessive (AA+AG vs. GG)			-	0.961
miR-124-1 rs531564 G>C				
GG	140 (72.9)	32 (74.4)	1.000 (reference)	
GC	44 (22.9)	10 (23.3)	1.043 (0.511 - 2.130)	0.908
CC	8 (4.2)	1 (2.3)	0.507 (0.068 - 3.794)	0.511
Dominant (GG vs. GC+CC)			0.942 (0.474 - 1.872)	0.865
Recessive (GG+GC vs. CC)			0.507 (0.069 - 3.734)	0.507
miR-126 rs4636297 G>A				
GG	131 (68.2)	32 (74.4)	1.000 (reference)	
GA	57 (29.7)	11 (25.6)	0.661 (0.329 - 1.328)	0.247
AA	4 (2.1)	0 (0.0)	-	0.948
Dominant (GG vs. GA+AA)			0.594 (0.296 - 1.192)	0.144
Recessive (GG+GA vs. AA)			-	0.950

AOR, adjusted odds ratio; LAD, large artery disease; HWE, Hardy-Weinberg equilibrium; 95% CI, 95% confidence interval; HR, hazard ratio. * Adjusted by age, sex, hypertension, diabetes mellitus, hyperlipidemia, and smoking. †*P*-value calculated cox proportional-hazards regression; *P*-values <0.05 are bold

Supplementary Table S8. Comparison of the six miRNA polymorphism frequencies for ischemic stroke SVD patient's survival.

Genotypes	Stroke patients (n=137)	Death patients (n=14)	Adjusted HR (95% CI)*	*P*†
miR-21 rs1292037 T>C				
TT	33 (24.1)	3 (21.4)	1.000 (reference)	
TC	69 (50.4)	6 (42.9)	0.387 (0.094 - 1.605)	0.193
CC	35 (25.5)	5 (35.7)	0.862 (0.186 - 3.987)	0.850
Dominant (TT vs. TC+CC)			0.439 (0.122 - 1.583)	0.211
Recessive (TT+TC vs. CC)			1.457 (0.430 - 4.938)	0.548
miR-21 rs13137 A>T				
AA	39 (28.5)	5 (35.7)	1.000 (reference)	
AT	76 (55.5)	7 (50.0)	0.308 (0.081 - 1.165)	0.084
TT	22 (16.1)	2 (14.3)	0.615 (0.093 - 4.052)	0.615
Dominant (AA vs. AT+TT)			0.370 (0.111 - 1.238)	0.109
Recessive (AA+AT vs. TT)			0.952 (0.180 - 5.028)	0.954
miR-26a rs7372209 C>T				
CC	79 (57.7)	8 (57.1)	1.000 (reference)	
CT	48 (35.0)	4 (28.6)	1.191 (0.333 - 4.261)	0.789
TT	10 (7.3)	2 (14.3)	1.372 (0.229 - 8.211)	0.731
Dominant (CC vs. CT+TT)			1.156 (0.383 - 3.490)	0.798
Recessive (CC+CT vs. TT)			1.330 (0.255 - 6.934)	0.736
miR-107 rs2296616 A>G				
AA	109 (79.6)	12 (85.7)	1.000 (reference)	
AG	27 (19.7)	2 (14.3)	0.589 (0.127 - 2.745)	0.503
GG	1 (0.7)	0 (0.0)	-	0.967
Dominant (AA vs. AG+GG)			0.586 (0.126 - 2.731)	0.498
Recessive (AA+AG vs. GG)			-	0.970
miR-124-1 rs531564 G>C				
GG	107 (78.1)	11 (78.6)	1.000 (reference)	
GC	28 (20.4)	3 (21.4)	1.047 (0.286 - 3.832)	0.945
CC	2 (1.5)	0 (0.0)	-	0.966
Dominant (GG vs. GC+CC)			0.930 (0.254 - 3.404)	0.913
Recessive (GG+GC vs. CC)			-	0.966
miR-126 rs4636297 G>A				
GG	86 (62.8)	9 (64.3)	1.000 (reference)	
GA	48 (35.0)	4 (28.6)	1.208 (0.353 - 4.128)	0.765
AA	3 (2.2)	1 (7.1)	0.993 (0.070 - 14.093)	0.996
Dominant (GG vs. GA+AA)			1.241 (0.403 - 3.828)	0.708
Recessive (GG+GA vs. AA)			1.363 (0.127 - 14.574)	0.799

AOR, adjusted odds ratio; SVD, small vessel disease; HWE, Hardy-Weinberg equilibrium; 95% CI, 95% confidence interval; HR, hazard ratio. * Adjusted by age, sex, hypertension, diabetes mellitus, hyperlipidemia, and smoking. †*P*-value calculated cox proportional-hazards regression; *P*-values <0.05 are bold

Supplementary Table S9. Comparison of the six miRNA polymorphism frequencies for ischemic stroke CE patient's survival.

Genotypes	Stroke patients (n=57)	Death patients (n=17)	Adjusted HR (95% CI)*	*P*†
miR-21 rs1292037 T>C				
TT	18 (31.6)	7 (41.2)	1.000 (reference)	
TC	25 (43.9)	6 (35.3)	0.806 (0.224 - 2.906)	0.743
CC	14 (24.6)	4 (23.5)	1.041 (0.237 - 4.571)	0.958
Dominant (TT vs. TC+CC)			0.849 (0.291 - 2.478)	0.766
Recessive (TT+TC vs. CC)			1.024 (0.316 - 3.315)	0.969
miR-21 rs13137 A>T				
AA	22 (38.6)	7 (41.2)	1.000 (reference)	
AT	22 (38.6)	7 (41.2)	0.542 (0.151 - 1.951)	0.351
TT	13 (22.8)	3 (17.6)	0.207 (0.028 - 1.537)	0.126
Dominant (AA vs. AT+TT)			0.540 (0.170 - 1.712)	0.298
Recessive (AA+AT vs. TT)			0.727 (0.200 - 2.643)	0.630
miR-26a rs7372209 C>T				
CC	24 (42.1)	8 (47.1)	1.000 (reference)	
CT	27 (47.4)	8 (47.1)	0.807 (0.270 - 2.416)	0.703
TT	6 (10.5)	1 (5.9)	**0.069 (0.005 - 0.909)**	**0.043**
Dominant (CC vs. CT+TT)			0.625 (0.229 - 1.709)	0.362
Recessive (CC+CT vs. TT)			0.260 (0.030 - 2.226)	0.221
miR-107 rs2296616 A>G				
AA	48 (84.2)	13 (76.5)	1.000 (reference)	
AG	9 (15.8)	4 (23.5)	1.784 (0.538 - 5.908)	0.346
GG	0 (0.0)	0 (0.0)	-	-
Dominant (AA vs. AG+GG)			1.784 (0.538 - 5.908)	0.346
Recessive (AA+AG vs. GG)			-	-
miR-124-1 rs531564 G>C				
GG	41 (71.9)	9 (52.9)	1.000 (reference)	
GC	14 (24.6)	6 (35.3)	1.580 (0.528 - 4.731)	0.416
CC	2 (3.5)	2 (11.8)	3.038 (0.500 - 18.474)	0.230
Dominant (GG vs. GC+CC)			1.855 (0.673 - 5.111)	0.235
Recessive (GG+GC vs. CC)			2.547 (0.491 - 13.219)	0.268
miR-126 rs4636297 G>A				
GG	41 (71.9)	11 (64.7)	1.000 (reference)	
GA	13 (22.8)	4 (23.5)	1.391 (0.384 - 5.042)	0.618
AA	3 (5.3)	2 (11.8)	2.850 (0.527 - 15.425)	0.227
Dominant (GG vs. GA+AA)			1.536 (0.534 - 4.420)	0.428
Recessive (GG+GA vs. AA)			1.914 (0.409 - 8.946)	0.412

AOR, adjusted odds ratio; CE, cardio-embolism; HWE, Hardy-Weinberg equilibrium; 95% CI, 95% confidence interval; HR, hazard ratio. * Adjusted by age, sex, hypertension, diabetes mellitus, hyperlipidemia, and smoking. †*P*-value calculated cox proportional-hazards regression; *P*-values <0.05 are bold
